# What is the effect of a Mediterranean compared with a Fast Food meal on the exercise induced adipokine changes? A randomized cross-over clinical trial

**DOI:** 10.1371/journal.pone.0215475

**Published:** 2019-04-18

**Authors:** Diana Silva, Rita Moreira, Marília Beltrão, Oksana Sokhatska, Tiago Montanha, Andreia Pizarro, Vanessa Garcia-Larsen, Rodrigo Villegas, Luís Delgado, Pedro Moreira, Joana Carvalho, André Moreira

**Affiliations:** 1 Basic and Clinical Immunology, Department of Pathology, Faculty of Medicine, University of Porto, Porto, Portugal; 2 Serviço de Imunoalergologia, Centro Hospitalar de São João E.P.E., Porto, Portugal; 3 Faculty of Nutrition and Food Sciences, University of Porto, Porto, Portugal; 4 Research Centre in Physical Activity, Health and Leisure—Faculty of Sports, Porto, Portugal; 5 Department of International Health, Johns Hopkins Bloomberg School of Public Health, Baltimore, Maryland, United States of America; 6 School of Public Health, University of Chile, Independencia 939, Santiago, Chile; 7 Epidemiology Research Unit- Instituto de Saúde Pública, Universidade do Porto, Porto, Portugal; Università degli Studi di Milano, ITALY

## Abstract

**Background:**

Adipose tissue-derived adipokines are pro-inflammatory cytokines involved in metabolic-related diseases and can be influenced by diet and exercise. We aimed to compare the effect of a Mediterranean (MdM) compared with Fast Food (FFM) meal on the exercise induced adipokines changes.

**Methods:**

In a double blinded cross over trial, 46 participants were randomly assigned to one of two standardized iso-energy pre-exercise meals: FFM or MdM-type. Three hours after each meal, participants completed a treadmill exercise test (EC). Serum adiponectin, resistin, PAI-1, lipocalin-2/NGAL and adipsin were determined by Luminex magnetic bead immunoassay. Wilcoxon signed rank test compared changes before/after meal and before/after EC and a linear mixed model evaluated the effect of meals on the adipokine response to exercise, adjusted for confounders.

**Results:**

Thirty-nine participants (mean age of 25, with a standard deviation of 5 years) completed the trial (56% females). For both interventions, a significant reduction of adipsin after each meal and a significant increase of lipocalin, PAI-1, adipsin and resistin, after exercise was observed. When exercise was preceded by a MdM meal a higher increase in adipsin levels was seen.

**Conclusion:**

Acute exercise induced an increase of circulatory levels of adipsin, resistin, lipocalin and PAI-1, but not adiponectin. A pre-exercise Mediterranean meal potentiated the increase of adipsin after the exercise test, which possibly relates to the immune regulatory role of adipsin. These changes suggest a cross-talk between the immune and metabolic immediate response to exercise and its modulation by the pre-exercise diet composition.

## Introduction

Unhealthy dietary intake and sedentary behavior in a genetically susceptible individual have been associated with adipokine dysregulation resulting both in adverse metabolic and immune responses [1]. Adiposity modulates inflammation and metabolic balance, both through adipokines, like adiponectin, as well as cytokines produced within the non-adipocyte tissue fraction, namely resistin [2]. These biomarkers might mediate the effect of obesity on other inflammatory diseases, namely in asthma[3]. This pro-inflammatory profile may be counteracted by exercise-induced fatness decrease[4]. Changes in dietary content, towards an increase of omega-3-fatty acid intake, induced an increase in adiponectin levels[5]. Taken together, evidence suggests that dietary habits and physical activity, in a long-term perspective, may modulate these adipokine influences on the metabolic and immune profile. Nevertheless, the effects of short-term interventions such as a single meal and an exercise bout remain poorly recognized.

Traditional Mediterranean diet is the dietary pattern that has been linked to a number of metabolic and health benefits, including reduced mortality risk and lower incidence of cardiovascular disease[6, 7] and reducing airway inflammation in asthma[8]. The benefits of even short periods of adherence to Mediterranean diet on inflammatory markers have been shown[9] and the adipose tissue inflammatory response, measured by IL-6, MCP-1, leptin and adiponectin gene expression[10], can be modulated by a single meal. A study comparing fast food with organic beef or turkey meal showed that the lesser the quantity of saturated and trans fatty acids, the higher the decrease in LDL-cholesterol [11].

Long-term aerobic and resistance training have also known metabolic and inflammatory positive effects [12]. Recently, it has been shown that higher intensity interval training seems to be more beneficial in improving cardiometabolic outcomes than moderate long-term exercise [13]. Adiponectin increased with exercise training both in adults[14] and children[15]. However, acute exercise did not show consistent results and only few studies have studied other adipose tissue-derived factors in the acute response to exercise[12]. A single exercise bout induces consistent immune changes [2] and increases serum inflammatory markers, particularly in the obese individuals [16]. Immune response to acute exercise may reflect a transient and time-dependent redistribution of immune cells to peripheral tissues, resulting in a heightened state of immune surveillance and regulation [17]. These beneficial effects might correlate with changes in the adipokine profile. Feeding status before moderate-intensity exercise influenced the adipose tissue adipokine gene expression, but did not affected circulating levels of adiponectin or leptin [18]. The effect of different meals on the adipokine response to acute high-intensity exercise has not been exhaustively studied. Therefore, we aimed to evaluate the effect of a Mediterranean (MdM) compared with a Fast Food (FFM) iso-energy meal on the acute exercise-induced adipokines changes.

## Methods

### Study design

**Fig 1:**
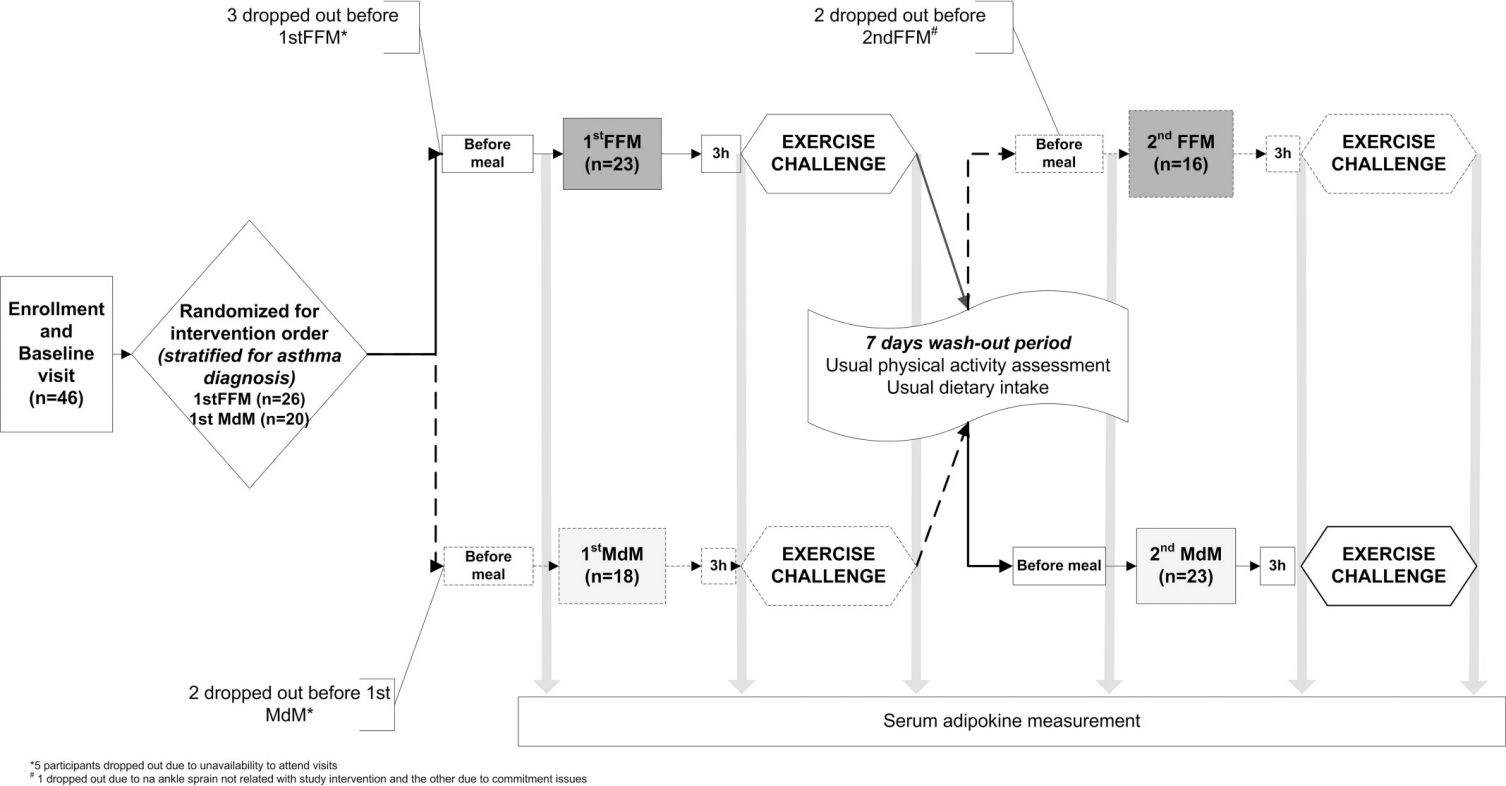
Flowchart of study participants.

This is a randomized crossover clinical trial, comparing the effect a high fat micronutrient poor meal, fast-food like meal(FFM) versus an iso-energy similar, micronutrient rich, Mediterranean like meal(MdM) in the immediate adipokine response to an exercise challenge(EC). A wash-out period of 7 days was performed after which participants crossed over to the alternate sequence (**Fig 1**). Study length was selected to reach complete normalization of post prandial inflammation and plasma lipid levels and avoid carry-over of the exercise response[19, 20].

Participants were evaluated at baseline for sociodemographic, lifestyle habits, as well as previous medical history. Physical activity and diet were assessed during the 7 days washing-out period. On the days of interventions participants were requested to have the same breakfast, avoiding caffeine and abstain smoking 12h before the meal. Main outcomes were evaluated immediately before, three hours after each meal and immediately after EC. This trial was previously registered in clinicaltrials.gov NCT02027675[21]. All procedures were approved by the ethics committee of Centro Hospitalar de São João, E.P.E. (CES 318–12) and written informed consent was obtained from the participants.

### Participants

Subjects aged between 18 and 35 years old were eligible and invited to participate through public advertising, accordingly to the previously described recruitment strategy [21]. Exclusion criteria were previously detailed and, briefly were: suffer from any major systemic disease[21]; pregnant or breastfeeding; under dietary restrictions or weight losing diet the last 3 months.

### Randomization, allocation and sample size

Participants were randomly assigned to the intervention order in a double-blinded fashion[21], stratified by asthma diagnosis. Outcomes were measured blinded to the participant’s allocation order.

Sample size was not specifically calculated to assess adipokine effect of meal-exercise challenge, as this was a secondary outcome of the study protocol. The power to evaluate a difference was calculated using liquid meal challenge differences for resistin in comparison with fasting [22], as no other previous studies have addressed the adipokine response to EC, assuming a minimal detectable difference in means of -0.6, a within-patient standard deviation of 1 with a sample of 40 participants the probability to detect treatment difference, using a paired sample t-test, at a two-sided 0.05 significance level will reach 74% power.

### Meal and exercise challenge

Meals were built to be energetically similar, respecting the Dietary Reference Intakes for macronutrient distribution. The FFM, consisted on a burger, BigMac, with medium size portion of French fries and a cola-drink and the MdM, consisted of vegetable soup, pasta, tomato, olive oil, herbs, garlic, bread, sardines, fruit and water. Design, preparation and detailed composition of the meals were previously detailed [21]. Meals nutritional composition are summarized in **S1 Table**. After each meal, participants were not allowed to eat or drink.

Exercise challenge was performed with a progressive multistage exercise testing, accordingly to Bruce Protocol, in a treadmill, until exhaustion [23]. Ventilation and respiratory gas analysis were measured via dynamic breath-by-breath measurement using the Oxycon Pro Metabolic Cart, Jaeger, Hochberg, Germany. There were no differences after FFM or MdM in each group for all the ventilatory, respiratory parameters and in median duration of the EC, as previously published [21].

### Adipokine and cortisol measurement

Blood was obtained by venipuncture from an antecubital vein and collected into two Terumo Venosafe serum-gel tubes 10 ml. Samples were centrifuged for 10 min at 400xg and the serum stored at -80°C. Adipokines were measured in serum, analyzed in duplicates using a bioassay multiplex technology, based on Luminex xMAP with a human adipokine panel (HADK1MAG-61K, EMD Millipore) that included adiponectin, resistin, PAI-1 (total), lipocalin-2/NGAL and adipsin, as previously described [24]. The intra-assay coefficients of variation(CV) ranged from 6% for adiponectin, adipsin and lipocalin to 10% for resistin and PAI-I. The inter-assay CV, evaluated in the repeated samples, ranged from 16% for adipsin, 26% for lipocalin, 27% for resistin and adiponectin and 29% for PAI-1.

Serum cortisol was measured in duplicate using a solid phase enzyme-linked immunosorbent assay (DRG Cortisol ELISA Kit) in the automatic ELISA Triturus analyser.

### Anthropometric, diet and physical activity evaluations

Height was measured using a portable stadiometer (SECA model 214). Weight and body composition using a digital scale Tanita BC-418. Usual dietary intake was assessed during wash-out period using a three-consecutive day food diary, two weekdays and one day during the weekend [25]. Nutrient analysis was performed using Food Processor, complemented with Portuguese Food Composition Table [26].

Physical activity was evaluated during the wash out-period in seven consecutive days by accelerometry (ActigraphWGT3Xmonitor) [21]. Troiano 2008[27] cut points were used. Data was presented as average time spent in moderate and in vigorous physical activity per day.

### Lung function, asthma and rhinitis diagnosis

Clinical asthma and rhinitis diagnosis were determined at baseline, as well as asthma pattern, accordingly to Global Initiative for Asthma recommendations [28] and diagnosis was established by a physician. Those individuals under regular asthma treatment were requested to maintain treatment. Lung function was evaluated by spirometry (Spirobank, Winspiro pro software, MIR) [29]. Asthma and rhinitis control assessed using Control of Allergic Rhinitis and Asthma Test presented a good overall control of the diseases.

Skin prick tests with common aeroallergens (*Dermatophagoides pteronyssinus*, grass pollen and weed pollen mixture, cat and dog epithelium, Alternaria) (Hal-allergy) were performed to define atopy if any positive test was found [30].

### Statistics

All statistical analyses were performed using the SPSS 24.0[31] or STATA 15[32] software. All graphics were made with GraphPad Prism 7. Normality of data distribution was assessed by the Kolmogorov-Smirnov test and QQ plot distribution. Results were presented as mean and standard deviation or as median and inter-quartile range, if data was not normally distributed, and in discrete variables as count and percentage. Count and percentage were analyzed with Pearson chi-square or Fisher exact test. For comparison between groups, non-parametric tests were used for paired samples Wilcoxon signed rank test was applied. For mixed model analysis, in data with skewed distributions (adiponectin, resistin and lipocalin) logarithmic transformation was done, as normal distribution was achieved analysis were made with this change. An analysis by intention to treat was planned. For missing data, Little´s MCAR test was performed, as missing completely at random was verified, a complete case analysis was done. Analysis for the carryover effect was performed using a two-way mixed ANOVA. A linear mixed model was performed with a random intercept for intervention order, and an autoregressive variance-covariance structure of order 1 used. The model was adjusted for the following confounders: age, gender, race, asthma diagnosis, BMI, FFM, smoking habits, moderate and vigorous physical activity, cortisol and diet. Data from the model was presented as marginal mean estimate with 95% confidence interval. Spearman correlations were performed to evaluate the relation between the several adipokine responses.

## Results

After recruitment strategy, from participants that met the eligibility criteria, 61 attended the screening visit. A total of 46 participants were included, of those 5 withdraw before any of the interventions and 2 before the second meal **(Fig 1)**. Baseline demographic and clinical characteristics of the participants are presented in **Table 1**.

**Table 1:** Baseline characteristics of the study participants.

	Total (n = 46)
Age years, mean(SD)	25 (5)
Female, n(%)	26(57)
Caucasian, n(%)	43(94)
Body mass index (BMI)	23.6[21.2;27.8]
Free fat mass (kg)	49.2[44.3;63.9]
Body fat (%), mean(SD)	22.3(7.2)
Smoking habits, active smokers, n(%)	7(15)
Atopy, n(%)	32(70)
Rhinitis diagnosis, n(%)	19 (41)
Asthma diagnosis, n(%)	13 (28)
CARAT score	24.5[23.0;29.0]
**Lung function**	
FVC %prev, mean(SD)	105(14)
FEV1%prev, mean(SD)	101(10)
FEV1/FVC %	104 [99;108]
FeNO,ppb	24 [15;47]
Cortisol(ng/ml)	144.0[100.1;193.0]
**Dietary Intake**	
Energy kcal	2392(586)
Carbohydrates, %TEV	51.2(6.6)
Protein, % TEV	16.7(3.4)
Lipids, % TEV	30.1(5.4)
Fiber, g/day	17.0[14.0;24.1]
**Physical activity**	
Average MVPA(min/day) mean(SD)	46.7(20.2)
Total time in sedentary activity (min) mean(SD)	2997(525)
Total time in moderate activity(min)] mean(SD)	241(110)
Total time in vigorous activity(min)	7 [0;37]

Data is presented as median and interquartile range [IQR], except if otherwise specified. MVPA- moderate vigorous physical activity; SD- standard deviation; TEV-total energy value

### Cortisol

No differences in serum cortisol changes existed between meals (MdM median -39.10 ng/ml 95% CI [-88.35;7.60] and FFM -31.75 [-81.13; -0.50]). An increase after exercise bout was seen, though being more pronounced after the FFM no difference between meals were observed (MdM 13.30 CI 95% [-17.05;65.40], p = 0.03 and FFM 32.90 CI 95% [-12.25;86.13], p = 0.02).

### Adipokines

**Fig 2:**
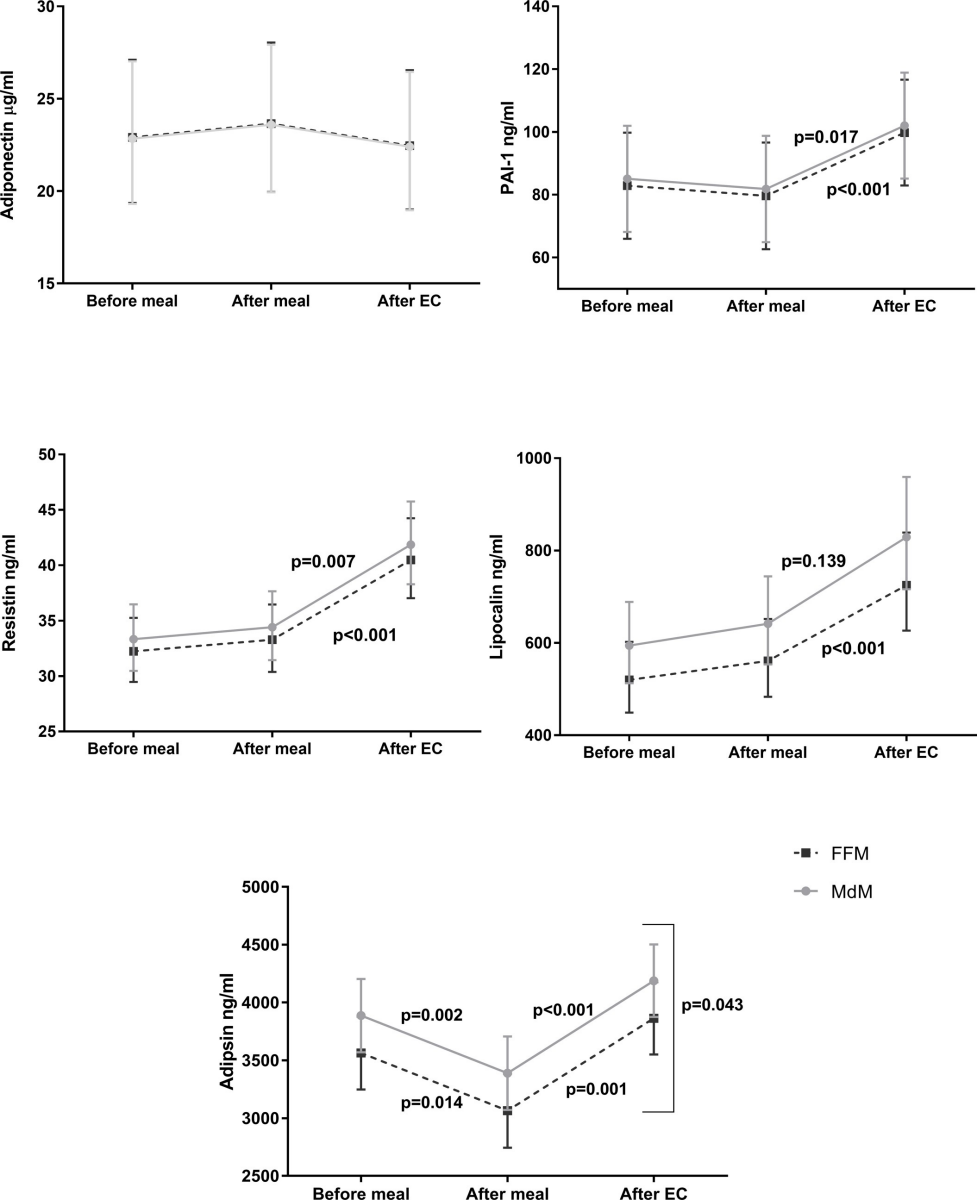
Points and connecting dots with error bar comparing Mediterranean and Fast Food meals effects on adipokine response to meal and exercise challenge using linear mixed model. Data is expressed in marginal mean estimate and 95% CI. EC- Exercise challenge.

No differences on the exercise induced adipokine changes existed between MdM and FFM meals using non-adjusted data (**Table 2**). When adjusted for confounders, MdM caused a significantly exercise induced increase of adipsin response (**Fig 2)**.

**Table 2:** Comparison between Mediterranean Meal(MdM) and Fast Food Meal (FFM) effects on serum adipokine response before and after each meal and after exercise challenge (EC) (n = 39).

								p Δ meals	p EC
	Before	After Meal	p	After exercise	p*	Δ Exercise-baseline	p#		
**Adiponectin (𝛍g/ml)**								0.971	0.429
**MdM**	19.86 [14.83;47.40]	18.57 [15.45;35.85]	0.390	20.75 [15.10;39.31]	0.536	0.89 [-2.50;2.42]	0.840		
**FFM**	20.98 [16.25;35.49	18.96 [15.51;35.51]	0.350	20.94 [15.73:33.01]	0.180	0.88 [-3.46;4.78]	0.301		
**Adipsin** **(ng/ml), mean (SD)**								0.814	0.168
**MdM**	3721.08(1342.67)	3353.26(1320.62)	**0.049**	4214.80(1849.98)	**<0.001**	493.72(1266.97)	**0.017**		
**FFM**	3600.09(1325.07)	3191.47(1123.51)	**<0.001**	3727.13(1256.79)	**<0.001**	127.04(905.11)	0.386		
**Lipocalin** **(ng/ml)**								0.587	0.833
**MdM**	541.90 [421.90;1015.86]	545.39 [401.10;1016.64]	0.953	887.04 [558.50;1149.74]	**0.007**	186.81 [-13.17;457.17]	**0.002**		
**FFM**	494.40 [364.61;858.3]	612.87 [358.67;957.89]	0.199	890.26 [523.93;1035.00]	**0.009**	184.67 [-37.10;509.96]	**0.001**		
**PAI-1** **(ng/ml), mean (SD)**								0.618	0.573
**MdM**	82.78(40.77)	80.56(42.31)	0.622	95.36(49.40)	**0.005**	12.58(28.61)	**<0.001**		
**FFM**	78.22(41.27)	78.28(44.61)	0.930	100.66(44.75)	**<0.001**	22.44(34.46)	**<0.001**		
**Resistin** **(ng/ml)**								0.811	0.777
**MdM**	35.59 [27.34;43.88]	36.23 [23.83;44.53]	0.683	38.14 [28.67;55.23]	**0.010**	7.51 [0.38;15.10]	**0.007**		
**FFM**	31.87 [24.19;41.86]	32.7 [25.70;39.94]	0.766	41.79 [3097;54.57]	**<0.001**	9.28 [0.53;20.33]	**<0.001**		

Data is expressed in median and interquartile range, except if otherwise specified. p values were performed using Wilcoxon signed rank test, except for adipsin and PAI-1 where paired sample t-test were used; SD-standard deviation

* between after and before exercise challenge(EC)

**#** between EC and baseline

Meals did not significantly affect the levels of adiponectin, resistin, lipocalin or PAI-1 (**Table 2**). After exercise, levels of resistin, lipocalin or PAI-1 increased (**Table 2**). When assessed with the linear mixed model, after adjustment for confounders, the effect of exercise remained significant, independently of the pre-exercise meal (**Fig 2)**. No carry-over effects were seen, and no interaction occurred between intervention order and time for all adipokine measured **(S2 Table)**.

Adipokines moderately correlated with each other in their response to the MdM (**Table 3**). When FFM was the intervention, adiponectin only correlated to adipsin and adipsin lost its correlation with lipocalin and resistin. Lipocalin and resistin responses were strongly and significantly correlated both in their response to each meal as well as to the exercise challenge (**Table 3**).

**Table 3:** Correlation coefficients matrix between adipokines responses to Mediterranean Meal(MdM) and Fast Food Meal(FFM) and exercise challenge.

	MdM					FFM				
	Adiponectin	PAI-1	Resistin	Lipocalin	Adipsin	Adiponectin	PAI-1	Resistin	Lipocalin	Adipsin
**Δ Meal**										
Adiponectin	**1**	0.496*	0.515*	0.438*	0.564*	**1**	0.286	0.197	0.075	0.530*
PAI-1	**-**	**1**	0.617*	0.477*	0.609*	-	**1**	**0.411**^**#**^	0.443*	0.458*
Resistin	**-**	**-**	**1**	0.796*	0.408*	-	**-**	**1**	0.789*	0.029
Lipocalin	**-**	**-**	**-**	**1**	0.437*	-	**-**	**-**	**1**	0.056
Adipsin	**-**	**-**	**-**	**-**	**1**	**-**	**-**	-	-	**1**
**Δ Meal-Exercise**										
Adiponectin	**1**	**0.359**^**#**^	0.259	-0.041	0.459*	**1**	0.25	0.481*	**0.342**^**#**^	0.584*
PAI-1	**-**	**1**	**0.336**^**#**^	0.004	0.29	-	**1**	**0.371**^**#**^	0.433*	0.134
Resistin	-	**-**	**1**	0.719*	0.422*	**-**	**-**	**1**	0.866*	0.589*
Lipocalin	-	-	**-**	**1**	**0.330**^**#**^	**-**	**-**	**-**	**1**	0.492*
Adipsin	**-**	-	**-**	**-**	**1**	**-**	-	**-**	**-**	**1**

*p<0.01

#p<0.05

When considering the response to an exercise bout, correlations were weaker and lost statistical significance after MdM-exercise challenge between adiponectin and resistin or lipocalin and between the associations of PAI-1 and Lipocalin or adipsin (**Table 3**). After FFM-exercise challenge adiponectin showed a mild correlation with resistin and lipocalin. No strong correlations were seen between adipokine and cortisol changes with exercise stimulus **(S3 Table)**.

## Discussion

Our study shows that a Mediterranean meal (MdM) may blunt the adipsin immediate response and potentiate its exercise induced increase in comparison with a fast food meal (FFM). MdM attenuated, non-significantly, the exercise induced cortisol increase. Exercise challenge(EC) induced an acute inflammatory response represented by an increase of lipocalin, PAI-1 and resistin independent of the previous type of meal. These findings highlight the importance of the pre-exercise dietary intake on both the immune and metabolic response to acute exercise.

Our findings are limited by a few reasons. Participants were normal weight, though obese or overweight were not excluded. Still, BMI and free fat mass were considered in the model. The number of individuals was insufficient to compare participants with or without asthma, although targeted by recruitment strategy, to minimize this effect, lung function was controlled. Furthermore, asthma control was assessed and the applied exercise stress test was not designed to induce exercise bronchoconstriction. A population with interest in achieving a healthier lifestyle could be more prone to engage in our trial and might have specific physical activity and dietary behaviors. Therefore, both characteristics were monitored and controlled. Despite the demanding protocol, attrition rate was below 20%. Previous studies were scarce to perform a sample size calculation accordingly to the adipokines assessed and it is possible that with a larger number of participants differences between meals would be reached, namely for lipocalin. It is not possible to exclude that a larger effect could be found in an obese population. The immediate response to exercise might differ in recreational versus elite athletes [33] Therefore, it is not possible to generalize between these populations. A graded exercise test was used instead of an exercise session, this choice allowed a better control of the challenge, although this type of test would be dependent on the cardiorespiratory fitness, this was controlled and pairwise comparison were performed. Adipokine changes might last until 4 hours after a meal [34], which might interfere with the EC. However, as we aimed to address the effect of a pre-exercise meal, a longer period would not allow to assess the targeted intervention and a shorter one would not induce a response [34]. Repeated evaluations could overcome these limitations and provide more information regarding the response through time, but this would escalate the complexity of the protocol and increase the drop-outs.

The evaluation of a meal-exercise intervention is one of the study strengths as it elucidates the response to a meal, but also to a stressor. The assessment of the effect of a MdM has rarely been addressed and this challenge provides a more real-life intervention, assessing the interactions between nutriments and not of a specific supplement. The study was controlled for main confounders, namely adiposity, physical activity, diet and cortisol. Another strength is the evaluation of adipokines with close relation with immune response, namely adipsin, but also PAI-1 and lipocalin which allows to monitor the cross-talk between immune and adipokine responses with exercise that probably exceeds the obesity-related inflammation.

Physical activity modulates the inflammatory cytokine response from the adipose tissue. Adipsin links the immune and adipose tissue, mediated by its activity as Factor D, a catalyzer of the alternative pathway of complement activation, being one of the major proteins expressed in the adipose tissue [35]. Recently, it has been shown to link fat cells and obesity to β-cell function, as adipsin increases insulin secretion mediated by the production of peptide complement 3a[36]. In our trial, a higher quantity of saturated fat was present in FFM, which is compatible with previous results that have shown a decrease in adipsin after a high fat meal [37]. In contrast to other studies, where a resistance exercise induced adipsin decrease [38], an increase after EC was observed. This may be explained by the use of an aerobic exercise bout. Although quantity of fat was similar between meals, type of fat, micronutrients, fibers and salt differed, it is possible that type of exercise and meal components modulated adipsin behavior.

A beneficial effect of high-intensity aerobic exercise training in adipokine profiles, has been suggested for overweight/obese women [39]. We failed to observe any significant adiponectin changes. However, our study differs in type of participants, exercise spectrum and on the pre-exercise meal. Nevertheless, our results were consistent with other findings where no changes after acute exercise were found [40]. Meals did not interfere with adiponectin levels, as occurred in a previous study comparing high versus low fat meals [41]. Therefore, although the quantity of a meal fat does not seem to have an impact, the type of fat might have a role, as in a three iso-energy meals challenge study, the one which was walnut-enriched lead to a higher postprandial adiponectin response [42]. A 4-week Mediterranean Diet reduced adiponectin levels [9]. A long term, one-year, randomized controlled trial lead to different results, as newly diagnosed type 2 participants that where adherent to the Mediterranean diet had lower C-reactive protein and higher circulating adiponectin levels[43]. Still, moderate correlations between adiponectin and other tested adipokines after MdM were seen.

Exercise challenge induced immediate changes in PAI-1, resistin and lipocalin. Though a MdM versus FFM did not modulated this response, lipocalin tended to have a different response after MdM. Lipocalin and resistin correlated strongly, which could be explained by their pro-inflammatory profile [44]. Resistin has potent proinflammatory properties [44] and has a role in glucose homeostasis, lipid metabolism and insulin action [15], it has showed to have an inverse association with levels of physical activity In a recent meta-analysis, regular exercise induced a decrease in resistin levels [15]. Our study differed, showing an increase, consistent with studies evaluating acute high-intensity exercise[45]. It is not possible to exclude the effect of the meal on resistin increase, although contradictory results occurred in the literature with similar meal tests, as outcomes were assessed at different time-points [22, 34]. Therefore, an inflammatory response to a strenuous stimulus is more likely.

Lipocalin-2 is a glycoprotein that has a role in the modulation of inflammation [46], acts in metabolic homeostasis and, as an acute phase protein, it has been use as potential biomarker in inflammatory diseases [46]. This adipokine has a role in the metabolic homeostasis being evaluated also in obesity-related metabolic diseases[46]. High-fat overfeeding did not induce any change in lipocalin-2 levels[47], similarly to our study. Consistent with previous reports[48], EC increased lipocalin levels. However, when adjusted for confounders after the MdM this difference lost significance. Despite no conclusions could be drawn for the effect of MdM, controlling both for age, gender, asthma diagnosis, physical activity and free-fat mass changed this relationship. As adipose tissue, namely fat mass quantity, might modulate the response to the different meals, studies with larger samples and in obese individuals might elicit different responses. Adipokines dysregulation appears to be associated with an increase of sympathetic nervous system activation[49]. Adiponectin altered gastric vagal afferent satiety signals in animal models[50]. Another axis is the potential modulation by exercise of the autonomic nervous system.

Acute stress, mediates sympathetic nervous system response and promotes increase of circulating PAI-1 [51]. PAI-1 inhibits fibrinolysis, is an acute-phase reactant and acts as an adipokine, as it is expressed in the adipose tissue. Two iso-energy meals that differed only on type of proteins showed a decrease on PAI-1 levels through time [52], a tendency also seen in our data. Another study, measuring the effect of an oral fat-challenge, did not found differences in PAI-1 [53]. EC promoted a significant response in PAI-1, independently of type of meal. Increased PAI-1 immediately after high-intensity EC have been seen in participants with untreated hypertension [54], although a decrease on PAI-1 activity was also reported[55]. This response, independent of fitness, in hypertensive [56], might be explained by the increased shear stress and blood pressure during exercise that correlates with the blood lymphocyte increase following acute exercise[17].

The mechanism of shear stress induced by acute exercise could explain the increase of other adipokines, namely lipocalin, which was already seen in combination of heat and exercise [57]. Furthermore, resistin release from adipose tissue is stimulated by steroid hormones [58], although no correlation between cortisol and resistin was seen.

In conclusion, a pre-exercise MdM potentiated the increase of adipsin after EC which might be related to the immune-regulatory role of adipsin. Acute exercise induced pro-inflammatory response to resistin, lipocalin and PAI-1, but not to adiponectin. This might represent a cross-talk between the immediate inflammatory and metabolic response to exercise, which could be addressed in other studies including the autonomic response.

## Supporting information

S1 CONSORT Checklist (DOC)

S1 Table Characterization of meals nutritional composition for Mediterranean Meal (MdM) and Fast Food Meal (FFM). (DOCX)

S2 Table Adipokine response accordingly to treatment sequence and carry-over effect analysis. (DOCX)

S3 Table Correlation coefficients matrix between cortisol and adipokines responses to Mediterranean Meal(MdM) and Fast Food Meal(FFM) and exercise challenge. (DOCX)

S1 Protocol Study protocol. (PDF)

## References

[1] Bays HE. Adiposopathy is "sick fat" a cardiovascular disease? J Am Coll Cardiol. 2011;57(25):2461–73. 10.1016/j.jacc.2011.02.038 . 21679848

[2] Nimmo MA, Leggate M, Viana JL, King JA. The effect of physical activity on mediators of inflammation. Diabetes Obes Metab. 2013;15 Suppl 3:51–60. 10.1111/dom.12156 . 24003921

[3] Zhang X, Zheng J, Zhang L, Liu Y, Chen GP, Zhang HP, et al Systemic inflammation mediates the detrimental effects of obesity on asthma control. Allergy Asthma Proc. 2018;39(1):43–50. 10.2500/aap.2018.39.4096 . 29279059

[4] Rossi FE, Diniz TA, Neves LM, Fortaleza ACS, Gerosa-Neto J, Inoue DS, et al The beneficial effects of aerobic and concurrent training on metabolic profile and body composition after detraining: a 1-year follow-up in postmenopausal women. Eur J Clin Nutr. 2017;71(5):638–45. 10.1038/ejcn.2016.263 . 28120855

[5] von Frankenberg AD, Silva FM, de Almeida JC, Piccoli V, do Nascimento FV, Sost MM, et al Effect of dietary lipids on circulating adiponectin: a systematic review with meta-analysis of randomised controlled trials. Br J Nutr. 2014;112(8):1235–50. 10.1017/S0007114514002013 . 25192422

[6] Dinu M, Pagliai G, Casini A, Sofi F. Mediterranean diet and multiple health outcomes: an umbrella review of meta-analyses of observational studies and randomised trials. Eur J Clin Nutr. 2018;72(1):30–43. 10.1038/ejcn.2017.58 . 28488692

[7] Liyanage T, Ninomiya T, Wang A, Neal B, Jun M, Wong MG, et al Effects of the Mediterranean Diet on Cardiovascular Outcomes-A Systematic Review and Meta-Analysis. PLoS One. 2016;11(8):e0159252 10.1371/journal.pone.0159252 27509006 PMC4980102

[8] Papamichael MM, Katsardis C, Lambert K, Tsoukalas D, Koutsilieris M, Erbas B, et al Efficacy of a Mediterranean diet supplemented with fatty fish in ameliorating inflammation in paediatric asthma: a randomised controlled trial. J Hum Nutr Diet. 2018 10.1111/jhn.12609 . 30378203

[9] Bedard A, Tchernof A, Lamarche B, Corneau L, Dodin S, Lemieux S. Effects of the traditional Mediterranean diet on adiponectin and leptin concentrations in men and premenopausal women: do sex differences exist? Eur J Clin Nutr. 2014;68(5):561–6. 10.1038/ejcn.2014.27 . 24595221

[10] Dordevic AL, Pendergast FJ, Morgan H, Villas-Boas S, Caldow MK, Larsen AE, et al Postprandial Responses to Lipid and Carbohydrate Ingestion in Repeated Subcutaneous Adipose Tissue Biopsies in Healthy Adults. Nutrients. 2015;7(7):5347–61. 10.3390/nu7075224 26140541 PMC4517001

[11] Bray GA, Most M, Rood J, Redmann S, Smith SR. Hormonal responses to a fast-food meal compared with nutritionally comparable meals of different composition. Ann Nutr Metab. 2007;51(2):163–71. 10.1159/000103277 . 17536194

[12] Gorgens SW, Eckardt K, Jensen J, Drevon CA, Eckel J. Exercise and Regulation of Adipokine and Myokine Production. Prog Mol Biol Transl Sci. 2015;135:313–36. 10.1016/bs.pmbts.2015.07.002 . 26477920

[13] Racil G, Coquart JB, Elmontassar W, Haddad M, Goebel R, Chaouachi A, et al Greater effects of high- compared with moderate-intensity interval training on cardio-metabolic variables, blood leptin concentration and ratings of perceived exertion in obese adolescent females. Biol Sport. 2016;33(2):145–52. 10.5604/20831862.1198633 27274107 PMC4885625

[14] Simpson KA, Singh MA. Effects of exercise on adiponectin: a systematic review. Obesity (Silver Spring). 2008;16(2):241–56. 10.1038/oby.2007.53 . 18239630

[15] Garcia-Hermoso A, Ceballos-Ceballos RJ, Poblete-Aro CE, Hackney AC, Mota J, Ramirez-Velez R. Exercise, adipokines and pediatric obesity: a meta-analysis of randomized controlled trials. Int J Obes (Lond). 2017;41(4):475–82. 10.1038/ijo.2016.230 28017965 PMC5382285

[16] Christiansen T, Bruun JM, Paulsen SK, Olholm J, Overgaard K, Pedersen SB, et al Acute exercise increases circulating inflammatory markers in overweight and obese compared with lean subjects. Eur J Appl Physiol. 2013;113(6):1635–42. 10.1007/s00421-013-2592-0 . 23361845

[17] Campbell JP, Turner JE. Debunking the Myth of Exercise-Induced Immune Suppression: Redefining the Impact of Exercise on Immunological Health Across the Lifespan. Front Immunol. 2018;9:648 10.3389/fimmu.2018.00648 29713319 PMC5911985

[18] Chen YC, Travers RL, Walhin JP, Gonzalez JT, Koumanov F, Betts JA, et al Feeding influences adipose tissue responses to exercise in overweight men. Am J Physiol Endocrinol Metab. 2017;313(1):E84–E93. 10.1152/ajpendo.00006.2017 . 28292758

[19] Baila-Rueda L, Mateo-Gallego R, Perez-Calahorra S, Lamiquiz-Moneo I, de Castro-Oros I, Cenarro A, et al Effect of different fat-enriched meats on non-cholesterol sterols and oxysterols as markers of cholesterol metabolism: Results of a randomized and cross-over clinical trial. Nutr Metab Cardiovasc Dis. 2015;25(9):853–9. 10.1016/j.numecd.2015.06.008 . 26232911

[20] Numao S, Katayama Y, Hayashi Y, Matsuo T, Tanaka K. Influence of acute aerobic exercise on adiponectin oligomer concentrations in middle-aged abdominally obese men. Metabolism. 2011;60(2):186–94. 10.1016/j.metabol.2009.12.011 . 20102772

[21] Silva D, Moreira R, Sokhatska O, Beltrão M, Montanha T, Garcia-Larsen V, et al Meal-exercise challenge and physical activity reduction impact on immunity and inflammation (MERIIT trial). Contemporary Clinical Trials Communications. 2018;10:177–89. 10.1016/j.conctc.2018.05.010 30009276 PMC6042468

[22] Gruendel S, Weickert MO, Garcia AL, Wagner K, Pfeiffer AF, Harsch I, et al Serum resistin increases in a postprandial state during liquid meal challenge test in healthy human subjects. J Endocrinol Invest. 2006;29(10):RC27–30. 10.1007/BF03349186 . 17185891

[23] Bruce RA, Blackmon JR, Jones JW, Strait G. Exercising testing in adult normal subjects and cardiac patients. 1963. Ann Noninvasive Electrocardiol. 2004;9(3):291–303. 10.1111/j.1542-474X.2004.93003.x . 15245347 PMC6932055

[24] Martos-Moreno GA, Burgos-Ramos E, Canelles S, Argente J, Barrios V. Evaluation of a multiplex assay for adipokine concentrations in obese children. Clin Chem Lab Med. 2010;48(10):1439–46. Epub 2010/06/26. 10.1515/CCLM.2010.276 . 20575747

[25] Abreu S, Santos PC, Moreira P, Santos R, Moreira C, Montenegro N, et al Predictors of adherence to the Mediterranean diet from the first to the second trimester of pregnancy. Nutr Hosp. 2014;31(3):1403–12. 10.3305/nh.2015.31.3.8158 . 25726240

[26] INSA. PortFir: National Health Institute Doutor Ricardo Jorge; 2017 [cited 2014]. A plataforma de informação alimentar em Portugal]. Available from: http://portfir.insa.pt/#.

[27] Troiano RP, Berrigan D, Dodd KW, Masse LC, Tilert T, McDowell M. Physical activity in the United States measured by accelerometer. Med Sci Sports Exerc. 2008;40(1):181–8. 10.1249/mss.0b013e31815a51b3 . 18091006

[28] Asthma GIf. Global Strategy for Asthma Management and Prevention2018. Available from: www.ginasthma.org.

[29] Miller MR, Hankinson J, Brusasco V, Burgos F, Casaburi R, Coates A, et al Standardisation of spirometry. Eur Respir J. 2005;26(2):319–38. 10.1183/09031936.05.00034805 . 16055882

[30] Heinzerling L, Mari A, Bergmann KC, Bresciani M, Burbach G, Darsow U, et al The skin prick test—European standards. Clin Transl Allergy. 2013;3(1):3 10.1186/2045-7022-3-3 23369181 PMC3565910

[31] Corp I. IBM SPSS Statistics for Windows, Version 24.0. Armonk, NY: IBM Corp; 2016.

[32] StataCorp. Stata Statistical Software: Release 15. In: Station C, editor. TX: StataCorp LP; 2017.

[33] Schwellnus M, Soligard T, Alonso JM, Bahr R, Clarsen B, Dijkstra HP, et al How much is too much? (Part 2) International Olympic Committee consensus statement on load in sport and risk of illness. Br J Sports Med. 2016;50(17):1043–52. 10.1136/bjsports-2016-096572 27535991 PMC5013087

[34] Yamauchi J, Osawa H, Takasuka T, Ochi M, Murakami A, Nishida W, et al Serum resistin is reduced by glucose and meal loading in healthy human subjects. Metabolism. 2008;57(2):149–56. 10.1016/j.metabol.2007.08.018 . 18191042

[35] White RT, Damm D, Hancock N, Rosen BS, Lowell BB, Usher P, et al Human adipsin is identical to complement factor D and is expressed at high levels in adipose tissue. J Biol Chem. 1992;267(13):9210–3. . 1374388

[36] Lo JC, Ljubicic S, Leibiger B, Kern M, Leibiger IB, Moede T, et al Adipsin is an adipokine that improves beta cell function in diabetes. Cell. 2014;158(1):41–53. 10.1016/j.cell.2014.06.005 24995977 PMC4128197

[37] Peake PW, Kriketos AD, Campbell LV, Charlesworth JA. Response of the alternative complement pathway to an oral fat load in first-degree relatives of subjects with type II diabetes. Int J Obes (Lond). 2005;29(4):429–35. 10.1038/sj.ijo.0802644 . 15111984

[38] Azizi M, Tadibi V, Behpour N. The effect of aerobic exercise training on β-cell function and circulating levels of adipsin in community of obese women with type 2 diabetes mellitus. International Journal of Diabetes in Developing Countries. 2017;37(3):298–304. 10.1007/s13410-016-0504-7

[39] Vardar SA, Karaca A, Guldiken S, Palabiyik O, Sut N, Demir AM. High-intensity interval training acutely alters plasma adipokine levels in young overweight/obese women. Arch Physiol Biochem. 2018;124(2):149–55. 10.1080/13813455.2017.1369998 . 28857629

[40] Ferguson MA, White LJ, McCoy S, Kim HW, Petty T, Wilsey J. Plasma adiponectin response to acute exercise in healthy subjects. Eur J Appl Physiol. 2004;91(2–3):324–9. 10.1007/s00421-003-0985-1 . 14586663

[41] Kennedy A, Spiers JP, Crowley V, Williams E, Lithander FE. Postprandial adiponectin and gelatinase response to a high-fat versus an isoenergetic low-fat meal in lean, healthy men. Nutrition. 2015;31(6):863–70. 10.1016/j.nut.2015.01.009 . 25933495

[42] Lozano A, Perez-Martinez P, Marin C, Tinahones FJ, Delgado-Lista J, Cruz-Teno C, et al An acute intake of a walnut-enriched meal improves postprandial adiponectin response in healthy young adults. Nutr Res. 2013;33(12):1012–8. 10.1016/j.nutres.2013.08.010 . 24267040

[43] Maiorino MI, Bellastella G, Petrizzo M, Scappaticcio L, Giugliano D, Esposito K. Mediterranean diet cools down the inflammatory milieu in type 2 diabetes: the MEDITA randomized controlled trial. Endocrine. 2016;54(3):634–41. 10.1007/s12020-016-0881-1 . 26860514

[44] Bokarewa M, Nagaev I, Dahlberg L, Smith U, Tarkowski A. Resistin, an adipokine with potent proinflammatory properties. J Immunol. 2005;174(9):5789–95. . 15843582 10.4049/jimmunol.174.9.5789

[45] Roupas ND, Mamali I, Maragkos S, Leonidou L, Armeni AK, Markantes GK, et al The effect of prolonged aerobic exercise on serum adipokine levels during an ultra-marathon endurance race. Hormones (Athens). 2013;12(2):275–82. . 23933696 10.14310/horm.2002.1411

[46] Abella V, Scotece M, Conde J, Gomez R, Lois A, Pino J, et al The potential of lipocalin-2/NGAL as biomarker for inflammatory and metabolic diseases. Biomarkers. 2015;20(8):565–71. 10.3109/1354750X.2015.1123354 26671823 PMC4819811

[47] Heilbronn LK, Campbell LV, Xu A, Samocha-Bonet D. Metabolically protective cytokines adiponectin and fibroblast growth factor-21 are increased by acute overfeeding in healthy humans. PLoS One. 2013;8(10):e78864 10.1371/journal.pone.0078864 24205333 PMC3799638

[48] Damirchi A, Rahmani-Nia F, Mehrabani J. Lipocalin-2: Response to a Progressive Treadmill Protocol in Obese and Normal-weight Men. Asian J Sports Med. 2011;2(1):44–50. 22375217 10.5812/asjsm.34821 PMC3289189

[49] Smith MM, Minson CT. Obesity and adipokines: effects on sympathetic overactivity. J Physiol. 2012;590(8):1787–801. 10.1113/jphysiol.2011.221036 22351630 PMC3573303

[50] Kentish SJ, Ratcliff K, Li H, Wittert GA, Page AJ. High fat diet induced changes in gastric vagal afferent response to adiponectin. Physiol Behav. 2015;152(Pt B):354–62. 10.1016/j.physbeh.2015.06.016 . 26074203

[51] Kaji H. Adipose Tissue-Derived Plasminogen Activator Inhibitor-1 Function and Regulation. Compr Physiol. 2016;6(4):1873–96. 10.1002/cphy.c160004 . 27783862

[52] Mortensen LS, Thomsen C, Hermansen K. Effects of different protein sources on plasminogen inhibitor-1 and factor VII coagulant activity added to a fat-rich meal in type 2 diabetes. Rev Diabet Stud. 2010;7(3):233–40. 10.1900/RDS.2010.7.233 21409315 PMC3061613

[53] Wu CJ, Yu ZR. Effects on blood glucose, insulin, lipid and proatherosclerotic parameters in stable type 2 diabetic subjects during an oral fat challenge. Lipids Health Dis. 2004;3:17 10.1186/1476-511X-3-17 15260879 PMC484204

[54] Gavriilaki E, Gkaliagkousi E, Nikolaidou B, Triantafyllou G, Chatzopoulou F, Douma S. Increased thrombotic and impaired fibrinolytic response to acute exercise in patients with essential hypertension: the effect of treatment with an angiotensin II receptor blocker. J Hum Hypertens. 2014;28(10):606–9. 10.1038/jhh.2014.18 . 24621621

[55] Cooper JA, Nagelkirk PR, Coughlin AM, Pivarnik JM, Womack CJ. Temporal changes in tPA and PAI-1 after maximal exercise. Med Sci Sports Exerc. 2004;36(11):1884–7. . 15514502 10.1249/01.mss.0000145447.61736.ed

[56] von Kanel R, Hong S, Pung MA, Mills PJ. Association of blood pressure and fitness with levels of atherosclerotic risk markers pre-exercise and post-exercise. Am J Hypertens. 2007;20(6):670–5. 10.1016/j.amjhyper.2007.01.005 . 17531926

[57] McDermott BP, Smith CR, Butts CL, Caldwell AR, Lee EC, Vingren JL, et al Renal stress and kidney injury biomarkers in response to endurance cycling in the heat with and without ibuprofen. J Sci Med Sport. 2018 10.1016/j.jsams.2018.05.003 . 29784554

[58] Adeghate E. An update on the biology and physiology of resistin. Cell Mol Life Sci. 2004;61(19–20):2485–96. 10.1007/s00018-004-4083-2 . 15526156 PMC11924563

