# Supplementary material for: What is the effect of a Mediterranean compared with a Fast Food meal on the exercise induced adipokine changes? A randomized cross-over clinical trial
Source: PLoS One. 2019 Apr 18;14(4):e0215475. doi: 10.1371/journal.pone.0215475 (PMC6472786; doi:10.1371/journal.pone.0215475)
Supplement: S1 Table — (DOCX) [file pone.0215475.s002.docx]

|  |  |  |
| --- | --- | --- |
|  | MdM^*^ | FFM^#^ |
| Energy (kcal) | 1070 | 1015 |
| Protein (g) | 45.0 | 31.0 |
| Protein (% TEV) | 16.8 | 12.2 |
| Carbohydrates (g) | 137.1 | 126.0 |
| Carbohydrates (%) | 51.3 | 49.7 |
| Sugars (g) | 31.3 | 52.0 |
| Fiber (g) | 23.0 | 7.0 |
| Fiber (% TEV) | 4.3 | 1.4 |
| Fat (g) | 37.9 | 43.0 |
| Fat (% TEV) | 33.2 | 37.7 |
| saturated (g) | 7.4 | 12.0 |
| saturated (% TEV) | 6.2 | 9.3 |
| MUFA (g) | 12.2 | NA |
| MUFA (%TEV) | 0.13 | NA |
| PUFA (g) | 8.4 | NA |
| PUFA (%TEV) | 0.09 | NA |
| n-3 (g) | 4.0 | NA |
| n-3 (%TEV) | 0.04 | NA |
| n-6 (g) | 2.3 | NA |
| n-6 (%TEV ) | 0.02 | NA |
| Trans (g) | 0.2 | NA |
| Trans (%TEV) | 0.17 | NA |
| Salt (g) | 3.0 | 2.9 |
| TEV- total energy value for the meal; NA- not available | | |

**Supplementary table 1.** Characterization of meals nutritional composition for Mediterranean Meal(MdM) and Fast Food Meal(FFM).
