# Supplementary material for: What is the effect of a Mediterranean compared with a Fast Food meal on the exercise induced adipokine changes? A randomized cross-over clinical trial
Source: PLoS One. 2019 Apr 18;14(4):e0215475. doi: 10.1371/journal.pone.0215475 (PMC6472786; doi:10.1371/journal.pone.0215475)
Supplement: S2 Table — (DOCX) [file pone.0215475.s003.docx]

**Supplementary table 2.** Adipokine response accordingly to treatment sequence and carry-over effect analysis.

|  | **Treatment period** | | | | | |
| --- | --- | --- | --- | --- | --- | --- |
|  | **MdM** | | | **FFM** | | |
| **Treatment sequence** | BM | AF | AE | BM | AF | AE |
| **Log Adiponectin** |  |  |  |  |  |  |
| **MdM then FFM** |  |  |  |  |  |  |
| Mean (SD) | 1.51(0.30) | 1.50(0.31) | 1.51 (0.37) | 1.50(0.30) | 1.43 (0.26) | 1.46(0.32) |
| Sample size | 15 | 15 | 15 | 15 | 15 | 15 |
| **FFM then MdM** |  |  |  |  |  |  |
| Mean (SD) | 1.27(0.33) | 1.27 (0.37) | 1.27(0.30) | 1.25 (0.29) | 1.25(0.29) | 1.30(0.33) |
| Sample size | 22 | 22 | 22 | 22 | 22 | 22 |
| **Adipsin** |  |  |  |  |  |  |
| **MdM then FFM** |  |  |  |  |  |  |
| Mean (SD) | 3533.85(1549.12) | 2906.12(1005.87) | 3948.84(1950.14) | 3590.45(1488.69) | 3034.34(1200.58) | 3500.85(1567.10) |
| Sample size | 15 | 15 | 15 | 15 | 15 | 15 |
| **FFM then MdM** |  |  |  |  |  |  |
| Mean (SD) | 3881.03(1268.28) | 3707.67(1486.76) | 4534.81(1851.42) | 3599.60(1273.30) | 3293.9(1085.29) | 3293.94(1085.297) |
| Sample size | 23 | 23 | 23 | 23 | 23 | 23 |
| Log Lipocalin |  |  |  |  |  |  |
| **MdM then FFM** |  |  |  |  |  |  |
| Mean (SD) | 2.78(0.26) | 2.76(0.31) | 2.88(0.27) | 2.66(0.31) | 2.74(0.37) | 2.83(0.30) |
| Sample size | 15 | 15 | 15 | 15 | 15 | 15 |
| **FFM then MdM** |  |  |  |  |  |  |
| Mean (SD) | 2.82(0.27) | 2.81(0.32) | 2.94(0.22) | 2.74(0.30) | 2.74(0.28) | 2.90(0.26) |
| Sample size | 23 | 23 | 23 | 23 | 23 | 23 |
| **PAI-I** |  |  |  |  |  |  |
| **MdM then FFM** |  |  |  |  |  |  |
| Mean (SD) | 79.94(42.99) | 76.98(40.90) | 94.02(46.73) | 75.53(42.80) | 64.81(41.18) | 92.40(36.69) |
| Sample size | 15 | 15 | 15 | 15 | 15 | 15 |
| **FFM then MdM** |  |  |  |  |  |  |
| Mean (SD) | 86.19(41.34) | 83.42(45.00) | 97.88(53.60) | 79.34(41.95) | 87.06(45.42) | 101.22(44.67) |
| Sample size | 23 | 23 | 23 | 23 | 23 | 23 |
| **Log Resistin** | | | | | | |
| **MdM then FFM** |  |  |  |  |  |  |
| Mean (SD) | 1.52(0.12) | 1.51(0.16) | 1.60(0.15849) | 1.4974(0.11255) | 1.5103(0.14524) | 1.5745(0.16921) |
| Sample size | 15 | 15 | 15 | 15 | 15 | 15 |
| **FFM then MdM** |  |  |  |  |  |  |
| Mean (SD) | 1.55 (0.15) | 1.54 (0.19) | 1.61(0.22) | 1.50 (0.15) | 1.50 (0.16) | 1.65(0.18) |
| Sample size | 23 | 23 | 23 | 23 | 23 | 23 |

Adiponectin analysis of carry-over effect: There was homogeneity of variances, as assessed by Levene's test of homogeneity of variance (p > 0.05).

The Box test was significant. Mauchly's test of sphericity indicated that the assumption of sphericity was violated for the two-way interaction. χ2(2) = 3.343, p =0 .008.There was no statistically significant interaction between intervention order and treatment time periods F( 2.725270; 95.384440)= 0.934003, p=0.420406, partial n2, E 2.472 using Greenhouse-Geisser.

Adipsin carry-over effect: There was homogeneity of variances, as assessed by Levene's test of homogeneity of variance (p > 0.05). There was not homogeneity of covariances. as assessed by Box's test of equality of covariance matrices (p = 0.042), nevertheless analysis was continued.Mauchly's test of sphericity indicated that the assumption of sphericity was violated for the two-way interaction, χ2(2) = 41.979, p < 0.001. Therefore, it was used estimate this adjustment and Greenhouse-Geisser. There was no statistical significant interaction between time and intervention order F(3.362, 12.033)=1.205, p=0.312 partial n2 0.032, using Greenhouse-Geisser.

Lipocalin carry-over effect: There was homogeneity of variances, as assessed by Levene's test of homogeneity of variance (p > 0.05). There was homogeneity of covariances. as assessed by Box's test of equality of covariance matrices (p = 0.104).

Mauchly's test of sphericity indicated that the assumption of sphericity was violated for the two-way interaction, χ2(2) = 30.221, p = 0.007. Therefore, it was used estimate this adjustment and Greenhouse-Geisser. There was no statistical significant interaction between time and intervention order F(3.919, 141.077)=0.242, p=0.911 partial n2 0.007.

PAI-I carry over effect: There was homogeneity of variances, as assessed by Levene's test of homogeneity of variance (p > 0.05). There was homogeneity of covariances, as assessed by Box's test of equality of covariance matrices (p = 0.168). Mauchly's test of sphericity indicated that the assumption of sphericity was met for the two-way interaction. χ2(2) = 15.083, p = 0.374. There was no statistical significant interaction between time and intervention order F (5.180)=0.833, p=0.258 partial n2 0.023.

Resistin carry-over effect: There was homogeneity of variances, as assessed by Levene's test of homogeneity of variance (p > 0.05). There was homogeneity of covariances, as assessed by Box's test of equality of covariance matrices (p = 0.919). Mauchly's test of sphericity indicated that the assumption of sphericity was violated for the two-way interaction. χ2(2) = 27.764, p = 0.016. Therefore, it was used the adjustment by Greenhouse-Geisser. There was no statistical significant interaction between time and intervention order F (3.713.133.681)=0.833, p=0.500 partial n2 0.037.
