# Supplementary material for: What is the effect of a Mediterranean compared with a Fast Food meal on the exercise induced adipokine changes? A randomized cross-over clinical trial
Source: PLoS One. 2019 Apr 18;14(4):e0215475. doi: 10.1371/journal.pone.0215475 (PMC6472786; doi:10.1371/journal.pone.0215475)
Supplement: S3 Table — (DOCX) [file pone.0215475.s004.docx]

|  | **MdM** | | | | | **FFM** | | | | |
| --- | --- | --- | --- | --- | --- | --- | --- | --- | --- | --- |
|  | Adiponectin | PAI-1 | Resistin | Lipocalin | Adipsin | Adiponectin | PAI-1 | Resistin | Lipocalin | Adipsin |
| **Δ Meal** | | | | | | | | | | |
| Cortisol | -0.104 | -0.087 | -0.002 | -0.146 | -0.046 | **0.353*** | -0.169 | -0.129 | **-0.356*** | 0.212 |
| **Δ Meal-Exercise** | | | | | | | | | | |
| Cortisol | 0.197 | 0.081 | -0.195 | **-0.327*** | -0.155 | -0.097 | -0.064 | -0.130 | -0.139 | -0.294 |
| *p<0.01; #p<0.05 | | | | | | | | | | |

**Supplementary table 3.** Correlation coefficients matrix between cortisol and adipokines responses to Mediterranean Meal(MdM) and Fast Food Meal(FFM) and exercise challenge.
