# Supplementary material for: What is the effect of a Mediterranean compared with a Fast Food meal on the exercise induced adipokine changes? A randomized cross-over clinical trial
Source: PLoS One. 2019 Apr 18;14(4):e0215475. doi: 10.1371/journal.pone.0215475 (PMC6472786; doi:10.1371/journal.pone.0215475)
Supplement: S1 Protocol — (PDF) [file pone.0215475.s005.pdf]

Department of Allergology and Clinical Immunology

São João Hospital Center

Faculty of Medicine Porto University

PHYSICAL ACTIVITY AND DIET AS DETERMINANTS OF IMMUNE FUNCTION

Study protocol

Diana Silva

Doctoral Program in Clinical Investigation and Health Services Research

10 December 2012

TABLE OF CONTENTS

PROJECT IDENTIFICATION 4

LIST OF PUBLICATIONS 5

SUMMARY 6

RESUMO 8

1.RATIONALE 10

2.BACKGROUND 13

3. RESEARCH QUESTION AND AIMS 14

4.METHODS 15

4.1. Participants and Study Design 15

4.1.1. Cross-cultural translation and validation of a respiratory infection symptom questionnaire (I) 15

4.1.2. Respiratory Infection susceptibility in athletes-illness prone profile (II) 18

4.1.3. Effect of Physical training on respiratory tract infection in the elderly (III) 20

4.1.4. Effect of an high fat meal on neuro-immune-endocrine response to an acute exercise challenge (IV) 23

4.1.5. Physical activity reduction effect on immune response (V) 27

4.2. Measurements 30

4.2.1. Respiratory infection symptoms (I, II,III) 30

4.2.2. Health state and quality of life measurements (I,II) 31

4.2.3. Nutritional Assessment (II, VI) 32

4.2.4. Airway Inflammation (III) 33

4.2.5. Lung function and airway responsiveness (III, IV,V) 34

4.2.6. Allergic and inflammatory systemic markers (III, IV, V) 34

4.2.7. Parasympathetic activity measure (III,IV) 35

4.2.8. Meal challenge (IV) 35

4.2.9. Exercise challenge (IV) 35

4.2.10. Immune-Endocrine and metabolomic mediators assessment (II, IV, V) 36

4.2.11. Physical activity measurement and intervention (IV,V) 37

4.3. Statistical analysis (I-V) 39

4.3.1.Cross-cultural translation and Validation of WURSS (I) 39

4.3.2. Respiratory Infection susceptibility in athletes and elderly (II,III) 40

4.3.3. Acute exercise and meal challenge (IV) 41

4.3.4. Reducing physical activity (V) 41

5. Safety 42

6. Ethical Considerations 46

Data Handling and Record Keeping 46

7. MILESTONES 48

8. TIMELINE 49

9. HUMAN AND MATERIAL RESOURCES 50

10. EXPECTED RESULTS AND OUTCOMES 51

Expected results 51
Outcomes 51
REFERENCES 52

ATTACHMENTS 57

Attachment 1 58
Attachment 2 59
Attachment 3 60
Attachment 4 61
Attachment 5 62
Attachment 6 63
Attachment 7 64
Attachment 8 65
Attachment 9 66

PROJECT IDENTIFICATION

Title

Physical activity and diet as determinants of immune function

A dieta e atividade física e o seu impacto no sistema imunológico

Authors

Diana Margarida G. S. Pereira da Silva

Supervisor: Professor Doutor André Moreira

Main scientific domain

Exercise Immunology

Secondary domain: Diet and neuro-immuno-endocrine modulation

Key-words

Exercise, immune function, immune-ssupression, diet, obesity, asthma

Project start and duration

From 1st September 2010 to 1st January 2015

LIST OF PUBLICATIONS

**I**	Cross-cultural translation and validation of a respiratory infection symptoms questionnaire
**II**	Upper respiratory tract infection susceptibility in athletes – a 3 months follow-up study
**III**	Effect of physical training on respiratory infections in the elderly
**IV**	Effect of an high fat versus healthy diet meal on neuro-immune-endocrine response to exercise
**V**	Effect of physical activity on immunity

SUMMARY

Background: Exercise is related to several acute and long-term changes in the immune system[1]. Respiratory infections have also been studied in relation to physical activity, being those with more sedentary behavior and the population that performs high intensity exercise more susceptible[2]. Moreover different characteristics interfere with this relationship, namely physical fitness, age and also diet. Indeed diet modulates immune functions and inflammatory processes [2, 3]. Specific populations namely obese and asthmatic have an intrinsic relation with this changes, but the exercise effect after a meal have never been studied in these particularly susceptible population[4, 5].

Main Problem: In a society where exercise is prescribed by physicians to increase physical condition, improve outcomes and reduce cardiovascular risk, the impact of exercise, acute or chronic, in the immune system is unknown. Specific populations can have different responses towards acute and chronic exercise and specific conditions like the type of ingredients ingested, namely fat meals, can be correlated with this responses. The true effect of exercise in the immune system is unknown.

Research question: Understand the effect of physical activity and diet as determinants of immune function.

Aims: Obtain a valid instrument to evaluate respiratory infections in Portuguese population; to find characteristics related to a illness-prone profile in high competition athletes during a bout of exercise; evaluate the impact of a moderate exercise program in a susceptible population (elderly); Correlate the effect of diet (high fat meal) in acute exercise response in a specific obese and asthma population; ascertain the physical inactivity impact on the immune system.

Methods:

Study design: An observational study with validation methodology will be applied to evaluate respiratory infections; in prospective cohort study of 12 weeks a group of athletes under high competition levels, respiratory infections and potential risk factors accordingly to literature will be evaluated; an prospective case-control with a moderate- exercise program intervention will compare the impact in respiratory infections in elderly under exercise training or not; a randomized, placebo controlled cross-over trial will evaluate the impact of a high fat/high energy meal challenge in an acute exercise challenge; randomized clinical trial will be used to ascertain the impact of physical activity reduction in the immune function

Selection of participants: For questionnaire validation will be selected participants with respiratory infections; athletes will be selected from a high elite pre-competition level team; elderly will be selected from general population, namely from senior universities and nursing homes; asthma and obese participants will be selected from an university population sample

Sample Size: 281 patients will be chosen as target for evaluation in respiratory tract infections validation; 40 patients will be evaluated in total in swimmers illness-prone study trial; 40 participants will be selected to evaluate exercise program in the elderly; 30 participants, with a

equal number of obese, asthma, obese and asthma and a control will be allocated to the meal interventions (60 participants in total) and a target of 60 participants will be randomized to the physical inactivity intervention.

Measurements: Upper respiratory tract infections number, severity and quality of life impact, immune system markers, complete blood cell count, pro-inflammatory and anti-inflammatory mediators, namely cytokines, and metabolomic.

Main Outcomes: Respiratory Infection Risk in Susceptible Population, Immune markers parameter related with acute exercise and physical inactivity interventions (balance between pro-inflammatory and anti-inflammatory cytokines).

Expected results: Obtaining a valid method for upper respiratory infections assessment, evaluate the risk factors related with respiratory infections in a athletes population, namely physical fitness, nutrition and stress showing the multifactorial side of immunessupression in athletes; risk of respiratory infections in a physical activity and inactivity intervention as well as immune mediators impact related with an acute exercise challenge and the interference with diet previously to the exercise. Demonstrate the effect of exercise in immune system in particularly susceptible population.

Research Team

Diana Silva, MD, resident in Allergy and Clinical Immunology in S. João Hospital Center;

Mariana Couto, MD, PhD student, resident in Allergy and Clinical Immunology in S. João Hospital Center

Ana Rita Moreira, nutritionist, PhD student in faculty of nutrition of Porto University

António Alberrto Macedo, MD, Family Physician, ULS Matosinhos

Joana Carvalho, PhD, associate professor in sports faculty of Porto University

Eduardo Arend, PhD student, licensee degree on sports science and physical education

Pedro Moreira, PhD in human nutrition, associate professor of human nutrition, vice-president of directive board of faculty of nutrition of Porto University

Sílvia Rocha, Assistant Professor, QOPNA, chemestry department , Aveiro University

André Moreira, MD, PhD Assistant Professor of Immunology, Faculty of Medicine, University of Porto; Medical Consultant of Immunoallergology, Centro Hospitalar São João, Porto

RESUMO

Introdução: O exercício está relacionado com alterações agudas e a longo prazo no sistema imune. Estudos anteriores relacionaram infeções respiratórias com a prática de exercício, estando as populações com comportamento sedentário e que realizam atividade física intensa mais susceptíveis. Além disso, diferentes características interferem com esta relação, nomeadamente aptidão física, idade e dieta. De facto, a dieta tem uma função moduladora dos processos imunes e inflamatórios. Populações específicas como obesos e asmáticos têm uma relação intrínseca com estas alterações, mas o efeito do exercício após uma refeição nunca foi estudado nestas populações mais susceptíveis.

Principal problema: na sociedade moderna, onde o exercício é prescrito para aumentar a condição física e diminuir o risco cardiovascular, o impacto do exercício no sistema imunitário é desconhecido. Populações específicas podem ter diferentes respostas em relação ao exercício agudo e crónico e condições específicas como tipo de ingredientes ingeridos, como refeições hipercalóricas, podem estar relacionadas com estas respostas. O verdadeiro efeito do exercício no sistema imune é desconhecido.

Pergunta de investigação: perceber o efeito da actividade física e dieta como determinantes da função imune.

Objectivos: Obter um instrumento válido para avaliar infeções respiratórias na população Portuguesa; encontrar características de um perfil predisponente à doença em atletas de alta competição durante o exercício; avaliar o impacto de um programa de exercício moderado numa população susceptível; correlacionar o efeito da dieta (hipercalórica) na resposta aguda ao exercício numa população específica de obesos e asmáticos; avaliar o impacto da inactividade física no sistema imune.

Métodos:

Desenho do estudo: um estudo observacional com metodologia de validação vai ser aplicado para avaliar infeções respiratórias; num estudo de coorte prospectivo de 12 semanas de um grupo de atletas de alta competição irão ser avaliadas infeções respiratórias e potenciais factores de risco de acordo com literatura; um estudo caso controlo prospectivo com intervenção de exercício moderado irá comparar o impacto das infeções respiratórias num grupo de idosos ; um ensaio randomizado, controlado com placebo estilo cross-over irá avaliar o impacto de uma refeição hipercalórica na resposta aguda ao exercício; um ensaio randomizado irá avaliar o impacto da redução da actividade física na função imune.

Participantes: para validação do questionário foram selecionados participantes com infeções respiratórias; atletas foram selecionados a partir de uma equipa de natação de alta-competição; idosos foram selecionados da população geral, nomeadamente universidades seniores e lares; participantes com asma e obesidade foram selecionados a partir de uma população universitária.

Tamanho amostra: 281 doentes foram escolhidos para avaliação da validação do questionário de infeções respiratórias; 40 doentes foram avaliados no estudo de nadadores de alta-competição para avaliar a propensão à doença; 40 participantes foram selecionados para avaliar o programa de exercício em idosos; 30 participantes, em grupos iguais de obesos, asmáticos, obesos e asmáticos e controlo serão alocados para a intervenção com refeição (60 participantes no total) e um alvo de 60 participantes serão randomizados para a intervenção da inactividade física.

Medidas: número de infeções respiratórias superiores, impacto na qualidade de vida, marcadores sistema imune, hemograma, mediadores pró-inflamatórios e anti-inflamatórios (citocinas, metaboloma).

Principais resultados: Risco de infeção respiratória em populações susceptíveis, parâmetros de marcadores imunes relacionados com exercício agudo e inactividade física (balanço entre citocinas pro-inflamatórias e anti-inflamatórias)

Resultados esperados: Obter um método válido para avaliar infeções respiratórias, avaliar os fatores de risco relacionados com infeções respiratórias em atletas (aptidão física, nutrição e stress) de forma a evidenciar o aspeto multifatorial da imunossupressão em atletas; risco de infeções respiratórias na atividade e inatividade física bem como o impacto nos mediadores imunes relacionado com uma prova de exercício agudo e interferência com dieta prévia ao mesmo. Demonstrar o efeito do exercício no sistema imune particularmente em populações susceptíveis.

1.RATIONALE

Physical activity is a generic term to any body movement, mediated by skeletal muscles activity and is performed by anyone in order to sustain life [6]. Exercise is frequently used interchangeably with physical activity, but implies an activity performed for a specific purpose such as improvement of physical condition or competition [6]. Acute and chronic exercise induces changes in both number and function of the immune system [1]. Also mucosa immunity, namely that mediated by specific IgA in saliva is depressed in periods of long and intensified training, which could be related to an increase susceptibility to infections[1]. The main focus of exercise and immune impact has been studied in the susceptibility to upper respiratory tract infections (URTI)[2].

Respiratory tract infections are very common and interfere with daily life. Diagnosis is clinical and, until recently, physician confirmation of an infection cause of the symptoms was considered the gold standard. Nowadays this paradigm has been questioned at light of the laboratory identification of pathogens [7]. The use of these techniques are sometimes expensive and time-consuming [1]. Surveys to access respiratory infections have been created, the first of all was the Jackson scale, and more recently the Wisconsin Upper Respiratory Symptoms Survey, which also has an evaluative illness-specific quality of life outcome instrument [8, 9]. These questionnaires have been proven useful to monitor respiratory infections in research conditions[10]. Still, no Portuguese version exists. This type of tool would enable physicians to monitor disease with patients at home and provide a long-term and distant monitoring of this condition and relate it with individual characteristics, physical activity and impact on their lives. Furthermore it would be useful to ascertain the relationship between specific risk factors and immune system susceptibility, namely exercise interventions.

A relationship has been proposed between respiratory tract infections and exercise proposed by a J-shaped model, still not supported by high quality of evidence [1, 2]. This model suggests that moderate exercise may lower the risk for URTI compared to sedentary individuals, but those under high intensity exercise and strenuous exercise periods may raise risk for URTI. It has been proposed a hypotheses that further factors should be considered in this model, namely physical fitness of the individual, concomitant allergy, inflammation, concomitant disease or infection, environmental factors and nutritional status [1, 2, 11]. The sum of these characteristics can define an illness prone profile, specific for each population.

Athletes are an especially susceptible population to respiratory infections, which have an important impact in their performance in high competition challenge. An "open window theory" of altered immunity has been used to explain this susceptibility[12]. After each bout of prolonged exercise it was demonstrated a period of 3 to 72 hours of altered immunity[13]. The acute exercise induces immune system responses, similar to those that occur in the infection period, increased neutrophils, monocytes, natural killer, and a higher concentration of stress hormones, namely cathecolamines, growth hormones and cortisol [4, 14]. Furthermore it enhances release of anti-inflammatory and pro-inflammatory mediators [15]. These changes

support the theory of increased susceptibility[4]. Nonetheless the true measure of immune function and its association with immune-supression is still an unmet need, as cited in the Walsh et al position paper [1]. The main issue is to understand if an athlete is an immune-compromised host and what is the true role of intense and prolonged exercise training in their susceptibility as well as other risk factors that might be involved, namely stress and nutrition[2, 3, 16, 17]. Athletes have reported less episodes of respiratory infections when under normal training comparing with their sedentary peers[3]. In a recent large study, in physically fit and active adults not in high intense training programs showed a reduction of the number and severity of respiratory infections[18].

Exercise and immune system changes are probably in relation with the subject susceptibility. Elderly are frequently recommended to perform physical activity and exercise due to its multiple positive effects on health-related problems[19] and it has been seen as a countermeasure to immune-senescence[20, 21]. It has been shown that the less physically active elderly have an increase on respiratory tract infections[22]. Most of the previous literature focuses on immune mediators response to exercise intervention in elderly, contradictory effects have been seen between cross-sectional and longitunal studies as it was reported by an recent thorough literature review [21, 23]. This suggests that perhaps other markers should be evaluated, namely specifically patient related outcomes. Only one study performed in postmenopausal women evaluated disease as a main outcome after a 12 months moderate exercise intervention and reported a reduced incidence of common colds[24]. Further investigation is needed to evaluate the true impact of chronic regular exercise in restoring immune function and which factors can be effective associated to potentiate it. Also, acute exercise might also have a deleterious effect and could limit the beginning of a regular physical activity in illness-prone subjects. This potential effect can be supported by the previously open window theory and immune changes related to them [13]. Acute exercise effect as seldom been studied in populations other then athletes or healthy physical active populations [12, 25, 26]. In Mills et al[27] study proposed that being physically fit is protective of inflammatory responses so the previously seen results might be more significant in sedentary and in persons with chronic inflammatory systemic diseases. Possibly, reducing the physical activity will be associated also with a decreased immune function response. This was previously demonstrated by recent studies of Pedersen group, where there was seen after a short reduction of physical activity a decrease in insulin sensitivity and cardiovascular fitness. Effect of this intervention on immune function has never been studied[28-30].

Populations like asthma patients, obese and asthma-obese also benefit from physical training and it is nowadays widely recommended [5]. Both populations are associated with an chronic inflammatory profile and an increase susceptibility to infections, namely respiratory tract infections[31, 32]. Especially in asthma it is questioned that this seen susceptibility is only an epiphenomenon, infections appear more severe in patients with underlying respiratory disease, or instead a reflection of altered host susceptibility[32]. No previous study has been performed in evaluating acute exercise challenge is this specific population, as epidemiologically the

patient related outcomes are more difficult to relate, there is a need to understand how inflammatory, endocrine and immune function markers behave with acute exercise. Nowadays new forms of inflammation and metabolic assessment appeared which are more informative, namely metabolomic and microbiome, these new mediators can open the knowledge of the exercise impact in this populations[33-35]. This project aims to further study the impact of acute exercise, using immune and new inflammatory biomarkers in these susceptible populations. As previously stated other aspects can be involved in the respiratory infection susceptibility, namely diet.

Dietary interventions have been mainly studied in athletes, namely to be used as an intervention to decrease exercise-induced immune-depression and also to improve performance[4]. High fat meal challenge has been recently studied in a obese asthmatic population and it showed an increase of airway inflammation, mediated by neutrophils and even a decrease in bronchodilator response[36]. In these susceptible populations it will be interesting to evaluate the effects of a high fat diet on post-exercise immunity. Through these results a specific recommendation can be posed to susceptible populations to start exercise activity and do exercise more safely and decrease respiratory infection susceptibility if a healthier diet is chosen. This project aims widen exercise immunology understanding skipping to an individual context susceptibility in order to understand expected results and make the future recommendations on how to prescribe exercise more safely.

2.BACKGROUND

Research has been intensified in athlete population to understand their respiratory infection susceptibility. In a systematic review by Moreira et al[2], it is agreed that moderate activity may enhance immune function, but high-intensity exercise temporarily impairs the immune competence. Still areas of controversy are suggested by this systematic review; relationship between exercise and upper respiratory infections is affected by poorly known individual determinants such as genetic, fitness, nutritional status and atopy. Mills et al compared physical fit and less fit individuals and showed that in acute response to exercise fit individuals had an attenuation of leukocyte-endothelial adhesion in response to exercise, suggesting a protected inflammatory response to exercise. Reduced physical activity intervention, has recently been used by Pedersen team to evaluate metabolic responses, and showed that this intervention reduced peripheral insulin sensitivity and body composition, furthermore it also showed that this short intervention reduced cardiovascular fitness evaluated by VO2 max [13, 28-30].

Relationship between physical fitness, diet, exercise and immune suppression has been mainly studied in athlete or highly physically active population as reported in the recent published systematic review of Gunzer et al[4]. No conclusion has been withdrawn due to limited research evidence on how to reduce post-exercise upper respiratory symptoms with single macronutrients, namely in high fat nutrition meals [4]. In asthma patients a recent randomizes study by Wood et al, non-obese subjects with asthma were compared with obese subjects who were submitted to a high fat meal challenge versus a low fat meal. It was shown that the participants allocated to the high fat meal showed 4h post-meal an increase in sputum neutrophil percentage, toll-like receptor 4 and a lower relationship of FEV1/FVC, thereby relating the fat consumed to an increase inflammation status. In another study by Meksawan, sedentary men (n=5) and women(n=6) had dietary fat intake during 3 weeks (19% calories and 50% calories) followed by an acute maximal exercise effort test. Evaluated inflammatory mediators of the immune system associated the bout of exercise to an increase of pro-inflammatory mediators which was aggravated by the fat meal for the previous 3 weeks in comparision with normal fat or high fat diet in this study the increase in pro-inflammatory mediators were not seen with a 50% caloric balance.

3. RESEARCH QUESTION AND AIMS

The research question of this project is: how physical activity and diet interfere with the immune system functions, namely respiratory infection susceptibility and illness prone profile.

The main outcomes are:

- Evaluate the use of a respiratory infections questionnaire to diagnose and characterize respiratory infections and their impact on lives in Portuguese patients with respiratory infections over 16 years old. Performa a cross-cultural translation and validation of WURSS-21;
- Evaluate the upper respiratory tract infections risk factors (demographics, fitness profile, inflammatory, metabolomic, neuro-endocrine and environment) in a population with a high level of exercise intensity;
- Test the effect of a controlled, moderate intensity long-term training program in an elderly population;
- Evaluate the effect of a high fat meal compared to an healthy diet meal on the neuro-immune-endocrine response to exercise;
- Study the effect of physical activity reduction on neuro-immune-endocrine mediators and respiratory infections.

4.METHODS

4.1. Participants and Study Design

4.1.1. Cross-cultural translation and validation of a respiratory infection symptom questionnaire (I)

This is a trans-sectional case control validation research project of a respiratory symptoms questionnaire to be applied in a three months follow-up period during the seasonal peak of upper respiratory infection from January to March 2012 in patients over 16 years old with and without respiratory infections.

Participants

All patients that require an unscheduled appointment due to acute illness in a primary health care center (USF Progresso, Perafita, Unidade Local de Sáude de Matosinhos) and from the Department of Allergy and Clinical Immunology from Centro Hospitalar de São João will be recruited. The inclusion criteria for respiratory infections cases are: over or equal 16 years of age, physician diagnosis of respiratory tract infections, with onset of symptoms within 48h and with Jackson score for respiratory infections over 3 or higher. In Jackson score eight symptoms (sneezing, nasal discharge, nasal obstruction, sore throat, cough, headache, malaise and chilliness) are classified as 0=absent, 1=mild, 2=moderate or 3=severe and presence of at least one of the first four cold specific symptoms is required for diagnosis. All those patients that attend to the primary care center or allergy department for other reason except respiratory symptoms/infections will be proposed to enroll the study project as controls. Exclusion criteria are: under 16 years of age, all patients with basal or throat symptoms that started more than 48h before they searched medical care. Results provided from patients with allergy and those without allergy complains will be compared for changes in respiratory symptoms score and in quality of life changes.

Procedures

The Wisconsin Upper Respiratory Symptom Survey (**Attachment 1**) is an empirically derived patient-oriented illness specific quality-of-life evaluative outcomes instrument. It is a validated questionnaire in English and is now translated into Spanish, Japanese, French, German, Korean, Russian, and Ukranian, with high correlation of previous questionnaires aimed to evaluate the presence of upper respiratory symptoms. It is also designed to reflect the impact of colds in quality of life and evaluate the severity through time [9, 37, 38]. This questionnaires correlate with laboratory assessed measures in a induced respiratory infections clinical trial[39]. This is a 21 items questionnaire, based on a 7 point Likert-type severity scale. To those will also be associated the items for headache, body-ache and fever [10, 40]. Questionnaire, WURSS-21 will be given to the included cases and controls, to be completed during 6 days and at the third

day it will be also filled in a validated questionnaire in Portuguese, MOS SF-36 (**Attachment 2**) [41, 42] of quality of life for convergent validity evaluation.
Demographic characteristics (age, gender, education, smoking habits, allergic diseases) will be collected when the questionnaire is delivered, written informed consent will be obtained if the patient decides to fill the questionnaire.

Cross-cultural translation

Cross-cultural translation will be provided by investigators that contact daily with patients with respiratory infections and follow their course. This procedure will be based in the previous published guidelines by Baiardini et al.[43] and all items will be included in the cross cultural translation process (namely questionnaire instructions, items and response choices). The linguistic validation will consist on three steps:

1. Forward translation (translate from source language, English, to target language, Portuguese) by two translators, native speakers in the target language and bilingual in the source language, supported by an experienced specialist in respiratory infection treatment and diagnosis; translators will independently produce a forward translation of the questionnaire, then a combined version, obtained by the agreement on a single reconciled version with all elements (translators and physician)
2. Backward translation (translation of the first reconciled forward version of the questionnaire back into the source language): requires a translator with no previous access to the questionnaire, native speaker of the source language and bilingual in the target language, this version will be sent to the authors for review and comment; then a comparison will be made with the original source version to detect any misunderstandings, mis-translations or inaccuracies in the forward version of the questionnaire
3. Patient testing (comprehension test): the final consensus version will be administered to a sample of 10 respondents, native speakers of the target language, over 16 years old, in a face to face interview, during which participants will be inquired if any difficulties were found in understanding the questionnaire and check the interpretation of all items; if any problem is encountered the interviewer should ask the person to propose alternatives and test alternative translations. This is aimed to determine whether the translation is acceptable, understood and if the language is simple/ appropriate. During interview, data will be collected: number of interviewed, age, difficulties and solutions. From this report a third and final version will be produced

Validation

Content validity of the Wisconsin questionnaire survey was previously studied in the validated versions of WURSS-44 and the short form WURSS-21[9, 38]. In the short form, will be assessed 7 different domains: global day severity, global change since yesterday, cough, throat, nasal, tiredness, functional/activity and other (head congestion). It will be also included

headache, body ache and fever to these domains, which has been used for WURSS-24 [10, 40].

Convergent validity will be assessed by comparison with a health related quality of life questionnaire, SF-36, already validated, which will be filed in at the third day of disease.

For criteria validity, the questionnaire will be compared between patients with respiratory infections and without respiratory infections diagnosed by physicians (which is the gold standard for respiratory infections diagnosis). Furthermore, inclusion criteria use the previously known Jackson criteria widely applied for respiratory infections diagnosis[8]. Both patients will fill WURSS during 6 days and SF-36 on the third day.

Reliability will be evaluated by the application of the same questionnaire through a 6 day period, first three days will be compared using internal consistency measures. As a respiratory infection symptom will change through time (patients will be asked if comparing to the previous day they feel that my cold is very much better, somewhat better, a little better, the same, a little worse, somewhat worse and very much worse) responsiveness to the questionnaire will be evaluated through already published methods[9, 44], namely Guyatt`s responsiveness index. This index demands the calculation of Minimal important difference, the mean value of day-to-day changes. Also the mean squared error of the scores will also be needed, which is based on those subjects reporting the same state.

4.1.2. Respiratory Infection susceptibility in athletes-illness prone profile (II)

This is a prospective, three month follow-up, cohort study to evaluate the risk factors for respiratory infection. A group of swimmers from a Porto sports team (Futebol Clube do Porto), from a high competition level and active during the follow-up period, which is coincident with a pre-competition season and higher incidence of respiratory infections (from January to March). During this time participants will be followed for respiratory infections episodes through a daily respiratory infections questionnaire, which is filled in when they began respiratory infections symptoms and then everyday during the scheduled-time illness. Acute respiratory infection number, duration severity of each episode will be characterized. All potential risk factors for respiratory infections will be evaluated at baseline and at the end of the study, except for urine analysis which will be collected every week and during the respiratory infection episode to establish an eventual association between changes of urinary metabolome in and outside the infection period.

Participants

All elite swimmers of Porto Sports Club from the main swimming team who are annually screened for asthma and atopy at Allergy Asthma and Sports Unit will be invited to participate in the project. The inclusion criteria will be: competitive level swimmer; aged above 16 year-old; either sex and of any race. A potential subject who meets any of the following criteria will be excluded from participation in this study: neurological or psychiatric illness which enable them to fill in the formulary/ and questionnaires and anything that, in the opinion of the investigator, would place the subject at increased risk or preclude the subject’s full compliance with or completion of the study. All these participants were previously included in the study, *“Mechanisms of airway damage in elite swimmers”* that was previously submitted and accepted in the Ethical Comission for Health of Centro Hospitalar de São João. For being included in this project written information about this follow-up will be provided and informed consent will be signed.

Procedures

Participants recruited in the previous project “Mechanisms of airway damage in elite swimmers” and all those that fill in the inclusion criteria, accept to participate and sign the informed consent will be evaluated at baseline.

In baseline the following characteristics and potential risk factors for respiratory infections will be screened:

- demographic data; full clinical history (namely asthma, respiratory or cardiovascular disease); chronic medication (any medication taken oral or inhaled); time of exercise practice and intensity of the trainings; immunization status and influenza vaccine; use of pro-biotics or vitamin supplements

- anthropometric and bio-impedance measures;
- nutritional state assessment and nutritional questionnaires (see description in measurements)
- sleep quality assessment questionnaires (**Attachment 3**) and anxiety symptoms questionnaire (Zung Self Rate Anxiety Scale- **Attachment 4**)
- lung function (espirometry), airway inflammation evaluated by induced sputum evaluation, airway hyper-responsiveness (methacholine provocation testing), bronchial challenge with inhaled capsaicin, exhaled nitric oxide measurement (*performed in the "Mechanisms of airway damage in elite swimmers" project-accepted by the ethical commission Centro Hospitalar de São João* **Attachment 5***)*
- cholinergic-parasympathetic system measure by pupillometry response (*performed in the "Mechanisms of airway damage in elite swimmers" project-accepted by the ethical commission of Centro Hospitalar de São João* **Attachment 5***)*
- blood withdraw for inflammatory cytokines measurement
- exhaled breath condensate, exhaled breath
- urine collection for metabolome measurements

During the follow-up it will be monitored through a respiratory infections questionnaire the episodes of respiratory infections, duration, severity and quality of life impact. A diary of all missed trainings will be kept during the follow-up period. Urine samples will be obtained every week and during the infections period.

Participants will also be submitted to a nutritional 24h dietary recall interview twice a month. The first one will occur at baseline and will be performed face-to-face with the investigator, so that the participant understand the purpose of the interview and also could be trained in the procedure. The following recalls will be assessed by telephone.

At the end of the follow-up period participants will perform:

- anthropometric and bio-impedance measures;
- nutritional state assessment and nutritional questionnaires;
- sleep quality assessment questionnaires and anxiety symptoms questionnaire (Zung Self Rate Anxiety Scale-- **Attachment 4**);

All variables will be assembled at the end of the study and individuals with respiratory infections during this period will be compared with those without the outcome. Associations will be performed *a posteriori* between urine metabolome analysis and the days with respiratory infections and before/after an infection episode.

4.1.3. Effect of Physical training on respiratory tract infection in the elderly (III)

*This study was previously presented and accepted by the ethical commission of Centro Hospitalar de São João (*__Attachment 6)__

The study was a non-randomized, un-blinded, controlled trial targeting patients over 60 years recruited by the Exercise Health for the Elderly program, coordinated in Sports Faculty of Porto University, from senior universities and nursing homes from Northern Portugal area. The selected participants performed a 36 week, 3 times per week, exercise-training program in the Sports University of Porto, with aerobic and anaerobic components using an individualized protocol by trained sports personal. During this period of time, respiratory tract infections were monitored through a symptoms diary and respiratory infections were defined as severe, moderate or mild accordingly to the symptoms assembly. Previously to the intervention a global physical condition was assessed (weight, height, heart rate, functional fitness and blood pressure were measured), co-morbidities were registered (hypertension, dyslipidemia, diabetes, ischemic heart disease, previous stroke, previous cancer diseases, ostheo-articular and osteoporosis), as well as smoking habits, influenza immunization status and if they previously practiced exercise.

Participants

Recruitment occurred from September until November 2010 through Exercise Health for the Elderly program, organized by The Research Centre in Physical Activity, Health and Leisure, at Faculty of Sport Sciences and Physical Education at the University of Porto, which receives patients from the Porto metropolitan area, Senior Universities from Porto and also nursing homes and daily care centers for the elderly in Northern region of Portugal. Participants were contacted by phone or personal contact at the universities or nursing homes. Intervention group participants belonged mainly from Porto metropolitan area, as they had the ability to travel to the training sessions that occurred at Faculty of Sport Sciences and Physical Education at the University of Porto. Control group were recruited from nursing rooms and daily care centers from Paredes de Coura, and all of those from Porto metropolitan area which were not able to participate in the intervention group by schedule incompatibilities. Participants were eligible if they had: over sixty years old, a medical report describing that they were physically able to practice aerobic and anaerobic exercise of moderate intensity and did not participated in previous physical exercise programs. Exclusion criteria included all patients with uncontrolled disease, namely hypertension, patients with cardiac or orthopedic prosthesis, patients with a psychological, familial or sociological problem that might compromise their compliance to the exercise program. Participants were provided with written information about the exercise training program and symptoms diary data collection. A total of 80 patients were recruited for

the study, 12 did not met the eligibility criteria and 30 refused to participate, so 38 patients were selected. Participants were then assigned to the training group and control group accordingly to the availability and proximity to the exercise training group.

Procedures

Exercise program

Exercise program was performed during 36 weeks and consisted in 3 times per week in non-consecutive days (Mondays, Wednesdays and Fridays). Each session was performed in group with all elements and included a 30 minutes aerobic training and 45 min of anaerobic training. Aerobic training consisted in 30 min walking at moderate intensity reaching 60-80% of the maximum heart rate (*monitored by polar team system, Finland*). Resistance or anaerobic training was performed during 45 min aiming 9 different muscular groups (leg press, chest press, leg extension, seated row, seated leg curl, abdominal flexion, low-back extension, biceps curl and triceps extension), each exercise was performed twice with 10 to 12 repetition at 60-80 % of repetition maximum (repetition maximum for each patient was previously calculated on base of *Epley formula*). Training was standardized, but adapted to each participant difficulties or handicaps, namely accordingly to previous osteoarticular conditions.

Intervention and control group were submitted first to a demographic, previous history of cardiovascular, osteoarticular, cancer or respiratory diseases, smoking habits, influenza immunization and history of previously practiced exercise. Baseline weight and height was also collected.

Respiratory Infections Symptoms Diary

For respiratory infections measure it was used a symptom diary, based in previously published symptoms diaries and common cold assessment questionnaires based in the Jackson index, Common Cold Questionnaire and Wisconsin diary symptoms short form-survey[45, 46]. The diary was built to answer to the specific demands of the population, namely age, real life conditions of the study as no patients with chronic previously reported respiratory diseases were excluded. Through this symptom diary it would be possible to quantify respiratory infections, their severity and duration. The questionnaire was subdivided in five main symptoms categories, similarly as occurred in the common cold questionnaire, but also including ear and eye symptoms. Furthermore allergy symptoms were included, namely nose and eye itching to exclude allergic symptoms in the evaluation of the symptoms diary. Patients were also requested to fill in if they had any symptoms and those symptoms reported in every and most of the days during the intervention study were excluded, as they were probably reported by patients with chronic respiratory diseases. The five domains were: general symptoms (shivering, muscle pain, tiredness, chest pain, dispnea), nasal symptoms (runny nose, obstructed nose, sneezing, nose itch), eye symptoms (itching eyes), throat symptoms (sore throat, itching throat, cough and hoarseness) and ear symptoms (ear pain) and body temperature. Temperature was

registered if the absolute value met above 37.5ºC. Each symptom would be classified accordingly to the severity in a likert scale from 0 to 7.

A pilot study was performed with an elderly population already in an exercise program and due to difficulties in filling the Likert scale it was adapted to classification of the symptoms when present in mild, moderate or severe.

All participants was given a symptom diary, and explained individually how it should be filled, also a written explanation was provided in the questionnaire itself. Symptoms diary should be filled daily if any of the symptoms was experienced. A contact to the investigator was provided if any questions or doubts were present. All participants were contacted telephonically each month to maintain compliance and questioned if they were still in the trial, if they had been sick or had any problem filling in the questionnaire. The questionnaire could also be filled by a third person if the participant was not able to read. In the nursing home the responsible for the elderly was also trained to know how the questionnaire should be filled.

4.1.4. Effect of an high fat meal on neuro-immune-endocrine response to an acute exercise challenge (IV)

This is a randomized cross-over trial, with a wash out period of 7 days, single-blinnded, placebo controlled. Study design is specified in figure 1. Participants will be recruited from advertisement and four different groups will be compared: obese-asthmatic; asthmatic non-obese; obese without asthma and non-asthmatic and non-obese. Stratification will be done accordingly to having asthma and BMI≥30kg/m2. All patients will be allocated to a meal challenge in which a high fat meal will be followed by a low fat meal or vice-versa, meal challenge order will be randomized and participants blinded to the intervention. Three hours after it will be followed by an exercise challenge, which is aimed to induce acute immune changes. The main outcome will be exercise-immune function (immune function markers) changes after a diet challenge. As secondary outcomes stress/inflammatory markers, endocrine mediators, as well as blood fatty acids will be measured. Lung function, VO2 and airway inflammation will be assessed by exhaled breath condensate inflammatory markers and eNO. Any adverse events of the challenge will be registered, namely respiratory infections, gastro-intestinal symptoms and muscular ache.

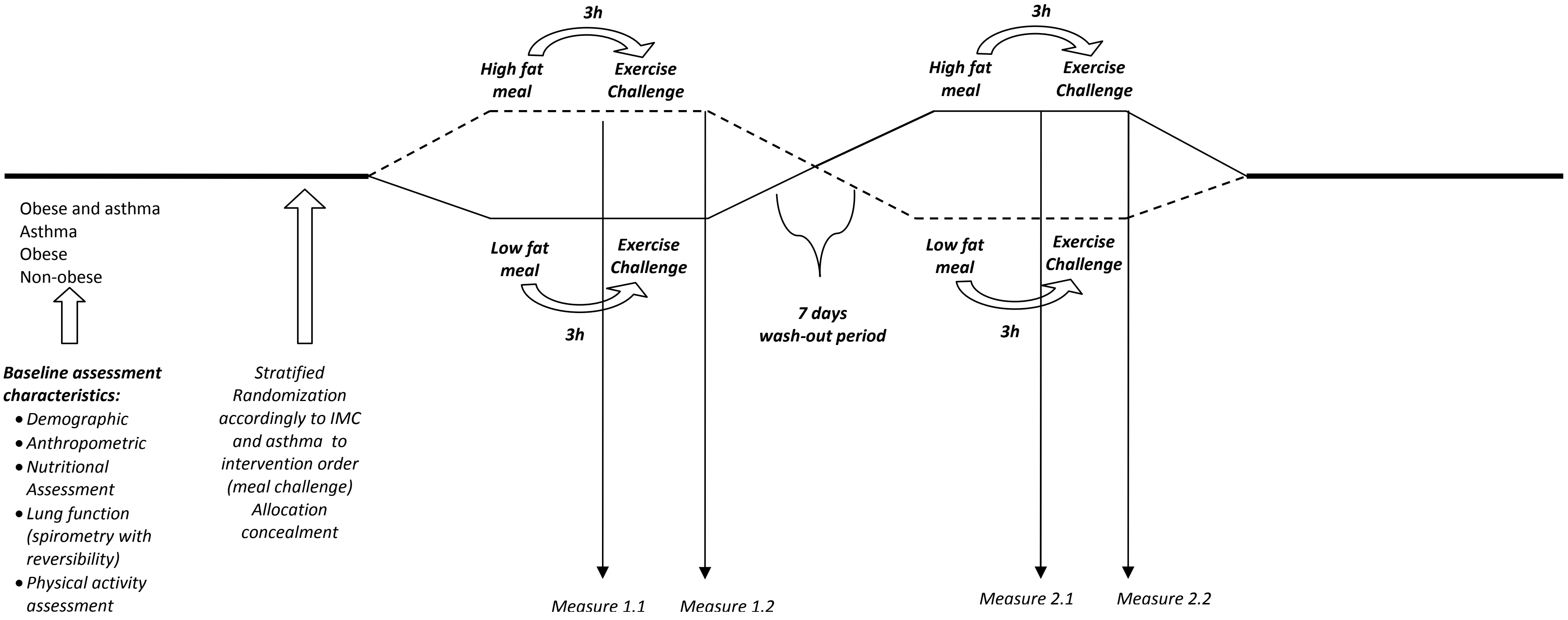

Figure1. *Randomized cross-over study trial, single-blinded to meal intervention. Measures (1.1; 1.2;2.1;2.2) immediately before and after exercise challenge include: Immune-endocrine-metabolomic response (blood, urine and saliva samples); Pupilometry; Lung-function and VO2 consumption; skin prick histamine reactivity*

Participants

Participants will be recruited from March to September 2013 by public advertisement in the Porto metropolitan Area from be from Porto University Faculties, by journal, media and through the academic organization information. In the advertisement a simple explanation will be provided about the study. Eligibility criteria will be: target age from 18 to 35 years old, have controlled asthma (which answered positively to the question have you ever been diagnosed with asthma by a medical physician? and controlled assessed by Asthma Control Questionnaire e o CARAT- **Attachment 7**), obese and non-obese and healthy participants for control group.
Groups will be stratified by having asthma and by their IMC in 4 groups: Asthmatic and obese; asthmatic non-obese; obese and non-asthmatic and non-obese. Patients will be considered as obese if they have BMI≥30kg/m2
Exclusion criteria will be: smoking in the past 6 months, abnormal electrocardiogram screening, pregnancy, diabetes mellitus, cardiac arrhythmia, angina, congestive heart failure, renal or hepatic failure and systemic disease, mal-absorption disease history, intestinal inflammatory disease, unable to adhere to full intervention or complete follow-up, unable to perform lung function testing and impaired mental status that makes difficult to follow or understand the instructions. Non-obese healthy controls need to have: no respiratory symptoms, had never a doctor`s diagnosis asthma, normal lung function and have to be steroid-naïve.

Procedures

After recruitment an interview will be performed to verify if patient verifies the inclusion criteria. This interview will assess weight and height, previous history of asthma and its control, presence of co-morbidities and availability to the study intervention and follow-up. If included a baseline assessment will be performed in the same day.
Baseline assessment will consist in obtaining information about: demographic characteristics, assessment of co-morbidities, anthropometric measure, blood pressure, rest heart rate, pupilometry, nutritional assessment, lung function measured by spirometry and bronchodilation, atopy assessed by skin prick test, basal physical activity assessment through a questionnaire, patients will also use an accelerometer for 7 days to evaluate their daily physical activity.

<u>Randomization and blinding</u>

Patients will be informed of the procedure but they will be blinded to which is the intervention (high fat or low fat meal).Stratified blocked randomization will be performed accordingly to being asthmatic or obese. This methodology was chosen despite acute changes are the aim, to exclude and evaluate the intervention order in the studied outcomes. Equal number of participants will be allocated to each group.
Allocation concealment will be prevailed as investigator will only know the intervention order for that participant at the moment the meal is provided. Exercise challenge and outcome assessment, namely urine, blood sample collection, exhaled breath condensate and lung function will only be performed by another investigator blinded to the meal order.

Intervention:

Meal challenge will be of high fat and low fat meal. Both meals composition will be controlled and built by a nutritionist team. All food left in the plate will be evaluated and registered to understand the caloric ingestion of each participant. No restrictions will be imposed previously neither diet control, as acute changes are the main outcome of this study and a daily diet diary will be provided. Only patients with controlled asthma can start the intervention, in the day of the first challenge they will return the accelerometer.

After the meal challenge, participants will not be allowed to eat or drink, in exception to water, and cannot perform any physical exercise. Then they will be taken to an exercise challenge laboratory, at Sports Faculty of Porto University, there 3 hours after the meal challenge blood, urine sample and exhaled breath condensate will be collected, heart rate at rest registered, skin test reactivity with histamine and spirometry performed. Exercise challenge, after the measurements 1 or 2 (figure 1) will be done. During the exercise heart rate response, exhaled VO2, CO2 and symptoms will be systematically registered. A physician and a cardiopulmonary technician will follow the challenge. Immediately after, a spirometry will be done and then at 5, 10 and 15 min after stopping the run, induced bronchoconstriction, pupillometry will be also measured and skin test with histamine and new blood and urine samples will be taken. If a fall in FEV1 over 10% occurs, bronchodilation will be done and patient treated. The exercise challenge will take at minimum 8 min, and patient will be asked to perform maximum effort, as the aim is to maintain for 4 min the heart rate at 95% of the target heart rate (220-rest heart rate).

Blood, exhaled air condensate, urine and saliva obtained before and after the exercise challenge will be used to analyze: metabolomic markers, immune function and inflammatory measures (in ex. leukocytes, lymphocytes, neutrophils, lymphocytes subpopulations and cytokine reduction, like IL-1, IL-6 and IgA in the saliva), stress mediators/hormones (ex. blood cortisol, growth hormone and epinephrine), endocrinological markers (ex. insulin, resistin, adiponectin, PAI-1, leptin) and fatty acids level (ex. monounsaturated, polyunsaturated and saturated fatty acids). Skin test sensitivity will be evaluate by histamine reactivity.

Patient will then go through a wash-out period of a week to avoid carry-over effect, as previously published intense acute exercise inducts changes in immune parameter that are maintained elevated for hours. It was also suggested an association between creatine kinase and IL-6 levels and the first can stay increased after exercise for almost 4 days after acute exercise challenge and by that the IL-6 level could be increase if shorter period of wash-out would be selected [14, 25].

4.1.5. Physical activity reduction effect on immune response (V)

This is a randomized controlled trial where participants, after a run-in period and eligibility evaluation, will be randomized to an intervention consisting in a reduction of physical activity by 50% or maintaining habitual physical activity for two weeks. Participants will be recruited from the same population as the meal-exercise challenge study and stratification will be done accordingly to having asthma and BMI≥30kg/m2 in order for both characteristics could be balanced between groups.

Participants will be at baseline characterized for their nutritional status, dietary habits and physical activity through questionnaire and basal assessment of all sports activity practiced during a week. Also generic quality of life questionnaire will be applied. A pedometer and accelerometer will be used to monitor physical activity. After this run-in period, if they have an adequate physical activity they will be allocated randomly to one of the interventions. Calculation of physical activity reduction will be performed individually, and a daily steps target will be demanded for each group. Both groups will use a pedometer during 3 weeks, except during the night or at circumstances that the device might be damaged (bath). They will be able to monitor if they achieved or exceeded the target number of steps for that day. Keeping the same regular diet without any new changes will be requested and new recall dietary assessment will be also performed in the end of the study. Immune function mediators will be the primary outcome to be assessed before and after the intervention period in both groups. As secondary outcomes endocrine and metabolomic evaluation will be performed.

Participants

Participants will be recruited from March to September 2013 by public advertisement in the Porto metropolitan Area from Porto University Faculties, by journal, media and through the academic organization information. Same participants recruited for the meal-exercise challenge trial will be invited to participate in the physical activity reduction. Eligibility criteria will be 18 to 35 years old, able to perform exercise. In this study a run-in period will be demanded to evaluate inclusion criteria. In all participants daily-life physical activity will be evaluated and use a pedometer for a 7 day period to evaluate the mean number of steps/day. Only those with more than 3500 steps per day will be included for randomization. Inclusion criteria will be: target age 18 to 35 years, healthy, nonsmokers, reveled no physical abnormalities and were physically active (performed >3500 steps/day). Groups will be balanced in the characteristics: having asthma and if they are obese (BMI≥30kg/m2) or non-obese.

Exclusion criteria will be: smoking in the past 6 months, pregnancy, diabetes mellitus, cardiac arrhythmia, angina, congestive heart failure, renal or hepatic failure and systemic disease, unable to adhere to full intervention or complete follow-up, impaired mental status that makes difficult to follow or understand the instructions.

Procedures

After recruitment an interview will be performed to verify if patient meets the inclusion criteria for study inclusion. This interview will assess weight and height, previous history of asthma and its control, presence of co-morbidities and availability to the study intervention and follow-up. If eligible, a run-in period will be performed that include: baseline assessment and a 7 week daily assessment of physical activity by a pedometer and accelerometer.

Baseline assessment will consist in: nutritional assessment, quality of life questionnaire (**Attachment 2**), asthma control questionnaire in asthmatic patients (**Attachment 7**), blood pressure, rest heart rate measurements, pupillometry, lung function measured by spirometry and bronchodilation, atopy assessed by skin prick tests, basal physical activity evaluated by Baecke (**Attachment 8**) and International Physical Activity questionnaire(**Attachment 9**). Physical activity will be also accessed by accelerometer and pedometer; only subjects that perform more than 3500 steps/day will be included. All other participants that are not eligible will receive dietary and physical activity written recommendations.

<u>Randomization</u>

Stratified blocked randomization will be performed accordingly to being asthmatic or obese. This methodology was chosen in order for both groups to be balanced for intervention comparison. Neither the participants or investigator team will be blind to the intervention procedure as there is a need to monitor number of steps/day to be compliant with the prescribed physical activity diary (keeping the same mean steps or reducing by 50% the number of steps/day). Equal number of participants will be allocated to each group.

Allocation concealment will be prevailed as investigator and participant will only know the intervention that they will be submitted at the second visit.

<u>Intervention:</u>

At baseline the included subjects will all collect blood samples, urine and exhaled air condensate. Then they will receive a list of indications adapted to their usual physical activity previously assessed in order for them to reduce by 50% their mean number of steps per day. As an example, they will be advised to: take the elevators instead of the stairs and riding car instead of walking or bicycling, reduce daily exercise training. The other participants will keep the same activity, also a number of target steps will be performed. Participants will not have knowledge of the mean number of steps that they usually performed, so both groups will receive specific written indications, but they will not know which will represent the intervention. No other contact will be done, but contact availability will be possible at anytime with investigators for any doubt. During the intervention the respiratory symptoms questionnaire will be given for them to fill in if they had an respiratory infection (**Attachment-1**)

After intervention all participants will deliver the pedometer. Blood, exhaled air condensate and urine will be collected. A questionnaire to evaluate nutritional assessment, quality of life (SF-36) and asthma control in patients with asthma will be performed.

The mediators that will be evaluated are: metabolomic markers, immune function and inflammatory measures (leukocytes, lymphocytes, neutrophils, lymphocytes subpopulations and cytokine reduction, IL-1, IL-6), endocrinological markers (insulin, resistin, adiponectin, PAI-1, leptin).

At the end of the study each patient will receive an individualized nutritional plan and physical activity indications performed by a nutritionist and a physician.

4.2. Measurements

4.2.1. Respiratory infection symptoms (I, II,III)

Data collection

Data will be collected throughout the study. In the validation study physicians will follow the inclusion criteria and each patient that meets possible inclusion for case or control, after accepting study inclusion demographic and clinical characteristics (age, gender, education, smoking habits, allergic diseases) will be collected before delivering the questionnaires. This information will be kept. The 6 days questionnaire of respiratory infections (**Attachment1**), and the SF-36(**Attachment 2**) and an pre-paid envelope with the Hospital/health center address will be given to the participant, explaining to him how it should be filled and that they could send it to the investigators by mail or left in the institution.

In the respiratory infection in athletes(I) a daily questionnaire will be provided and participants will fill in the questionnaire if they fill sick and daily until the symptoms have ceased. At the end of the 3 months follow-up questionnaires will be retrieved.

In the elderly population a simplified symptoms diary was provided, where they everyday coated which of the respiratory symptoms they had.

Respiratory infections symptoms questionnaire (I, II)

Respiratory infections symptoms questionnaire has 21 items, organizes in a 7 point Likert-type severity scale (from 1, very mild to 7, severe and one 0, without symptoms). Two items are used to classify the global-severity-today (how sick do you feel today and compared to yesterday I fell my cold is…). The severity score includes 10 items classifying in the last 24h respiratory symptoms (runny nose, plugged nose, sneezing, sore throat, scratchy throat, cough, hoarseness, head congestion, chest congestion, feeling tired) and 9 items of quality of life impact (think clearly, sleep well, breath easily, walk, climb stairs, accomplish daily activities, work outside home, work inside home, interact with other, live your personal life) [10, 40].

An acute respiratory infection episode is defined by having all these items for at least 2 days:

- answering "Yes" to either: "Do you think you have a cold?" or "Do you think you are coming down with a cold?"
- reporting at least 1 of 4 cold symptoms (or synonyms) (nasal discharge (runny nose); nasal obstruction (plugged or congested); sneezing; or sore (scratchy) throat)
- scoring at least 2 points on the Jackson scale (Jackson score is calculated by summing 8 symptom scores (sneezing, headache, malaise, chilliness, nasal discharge, nasal obstruction, sore throat and cough), rated as 0=absent, 1=mild, 2=moderate, and 3=severe.

From the first day of acute respiratory infection day and forward each participant fills out a daily WURSS until they answer "No" to the question "Do you think that you are still sick with this respiratory infection?" for 2 days in a row. The last day the participant answers "Yes" and fills out a WURSS instrument will be the last day classified as acute respiratory infection.

Daily WURSS summary scores are calculated by summing scores of individual item scores, excluding the first and last items, which have categorically different reference domains, and are analyzed separately. Summary scores of ARI illness episodes are calculated by summing daily scores.

Respiratory infections symptoms diary (III)

Symptom diary was built accordingly to the previously published respiratory infection assessment and after a pilot study to search for comprehensibility and adaptability to the population age [45, 46]. It was subdivided in five main symptoms categories: general symptoms (shivering, muscle pain, tiredness, chest pain, dispnea), nasal symptoms (runny nose, obstructed nose, sneezing, nose itch), eye symptoms (itching eyes), throat symptoms (sore throat, itching throat, cough and hoarseness) and ear symptoms (ear pain) and body temperature. Temperature was registered, if the absolute value was above 37.5ºC. Each symptom would be classified accordingly to the severity in mild, moderate or severe by the participant.

Respiratory infections were defined accordingly to symptoms assembly and period of time. Duration of respiratory infection was calculated accordingly to the symptoms that were correspondent to infection episode duration. Severity of the respiratory infections was classified as mild, moderate or severe, based in the type of symptoms and if they affected general health state of the individual.

Probable respiratory infection was defined by obtaining in at least three consecutive days, one of the four different settings:

- moderate or severe symptoms in two of five symptom categories (excluding the allergic symptoms namely nasal or ocular pruritus);
- mild/moderate symptoms in more than 3 symptom categories
- cough associated to at least 3 symptoms of 2 different symptom categories
- fever with other respiratory symptom.

Severity of respiratory infection was classified in: severe (participant had a systemic infection and/or severe local symptoms); moderate (mild systemic symptoms with moderate local symptoms) and mild (mild to moderate local symptoms).

4.2.2. Health state and quality of life measurements (I,II)

Health state and quality of life questionnaire (MOS SF-36v2)

At the third day of illness in the respiratory infection questionnaire validation it will be also filled in a validated questionnaire in Portuguese, MOS SF-36 (**Attachment2**), of health state. Quality of life was assessed using the SF-36, a self administered, widely used questionnaire, and validated to quantify subjective reports of health in terms of functional status, emotional status, and general well being[41, 42]. This generic instrument is composed of 36 questions, distributed

into eight different dimensions of health: physical function, role limitations related to role physical, bodily pain, general health perception, vitality, social functioning, role limitations due to emotional problems, and mental health. Each dimension is reported on a scale from 0 to 100 with a higher score reflecting better quality of life[41, 42].

Anxiety questionnaire

The Zung self-rating anxiety scale is a questionnaire aimed to evaluate a state of anxiety more than a anxious personality[47]. It has been validated in portuguese and is composed by 20 items that explore affective (5 items) and somatic (15 items) aspects of anxiety, on a 4-level scale[47, 48]. The total point score can range from 20 to 80. A high score indicate a high degree of anxiety. (**Attachment 4**).

Sleep and sleep quality questionnaire

To understand and evaluate sleep quality a questionnaire will be applied, asking sleep habits and sleep quality, both addressed by the Pittsburgh Sleep Quality Index. This is a self-rated validated questionnaire that assesses sleep quality and sleep disturbances over a 1- month time interval. A score >5 is used to define a ‘poor sleeper’. (**Attachment 3**)

4.2.3. Nutritional Assessment (II, VI)

Anthropometric measures (II,IV)

Anthropometric measures will include weight, height and specific measures to determine body composition, namely a triceps skinfold thickness and medial calf skinfold thickness.

Weight will be measured to the nearest 0,1 kilogram and height will be measured to the nearest 0,5 centimeters. Subjects will be lightly clothed and bare-footed.

After height and weight, it will be calculated body mass index (BMI) - weight (kilograms) divided by square of height (meters).

Individuals will be classified according to World Health Organization (WHO) classifications: normal weight, 18.5 to 24.9 kg/m^2; overweight, 25.0 to 29.9 kg/m^2; obesity, 30.0 kg/m^2 or plus.

Skinfold thickness measurement will be taken using a slim guide skinfold calipers and according to procedures described by International Society for the Advancement of Kinanthropometry (ISAK)[49]. Three measurements will be taken, at each site (+- 0,1 mm). Percentage body fat will be calculated using prediction equations (Males: % body fat = 0,735 * (triceps + calf) + 1.0 / Females: % body fato = 0,610 * (t+c) + 5.1), as previously published [50].

Waist circumference will be measured according to procedures described on ISAK[49]. Individuals will be classified in abdominal overweight or obese, according to the following sex-specific cut-offs: females - abdominal overweight 80 - 88 cm, abdominal obesity 88 cm or plus; males - abdominal overweight 94 - 102 cm, abdominal obesity 102 cm or plus[51].

Dietary Reports (II, IV)

Dietary intake will be assessed by a repeated 24-hour recall. Participants will be asked to provide detailed information about their dietary intake, through a 24-hour dietary recall method. This method will be conducted in four steps: first, individuals will be asked to inform the interviewer about all foods and beverages they've consumed in the previous 24 hours; second it will be questioned for foods that are documented as being frequently forgotten; third, individuals will be asked about the time and occasion at which foods were consumed; finally, the interviewer will ask questions about food description and amounts.

Participants of study IV, will provide their reports on the first and second meetings.

4.2.4. Airway Inflammation (III)

Sputum collection and analysis will be performed as described previously in literature [52]. Patients will perform previously bronchodilation with salbutamol followed by a protocol of inhalation of hypertonic saline (5%NaCL) generated by an ultrasonic nebulizer during 5 min periods for 20 min. Sputum will then be obtained. After the sample is collected it will be selected in a Petri dish, and the selected sample treated with dithiothreitol (Sputolysin; Calbiochem Corporation, San Diego, CA, USA). The suspension will be centrifuged and the cell pellet re-suspended. Cytospins will be prepared and stained using May-Grünwald/Giemsa. Differential cell counts will be made by counting a minimum of 500 nonsquamous cells. Sputum eosinophilia will be defined as a sputum differential of eosinophil count over 4%.

Metabolomics is the study of small molecules (0,1 kDa) generated from cellular metabolic activity and it seems to be a potential biomarker for airway inflammation[33]. Exhaled breath has potential to be a matrix with relevant metabolomic information to characterize asthma[34, 53]. Comprehensive two-dimensional gas chromatography–time of flight mass spectrometry (GC ×GC–ToFMS) has been used to on exhaled breath in patients with asthma and a pattern of six compounds belonging to the alkanes were found that characterized the asthmatic population: nonane, 2,2,4,6,6-pentamethylheptane, decane, 3,6-dimethyldecane, dodecane, and tetradecane. This profile can be useful for distinguishing patients with asthma from other different inflammatory airway disease and possibly new profiles can be withdrawn from this technique, namely characterizing an illness prone profile [34, 53]. The breath sampling will be obtained as previously published [34, 53]. Briefly, in a previously clean 1 L Tedlar® bags and after washing the mouth with water will exhale deeply into the Tedlar® bag previously holding their breath for 5 seconds. All bags will be analyzed for a maximum of 6 h after the collection [34, 53]. This analysis can also be performed in exhaled air condensate, this is obtained accordingly to the previous published guidelines. Extraction/concentration will be done with HS-SPME methodology to coating fiber, this will then be introduced into the GCx GC–ToFMS, which consists of an Agilent GC 7890A gas chromatograph, with a dual stage jet cryogenic modulator. Detection will then be performed by a high-speed ToF mass spectrometer. In order

to identify the different compounds, the mass spectrum of each compound detected will be compared to those in mass spectral libraries databases[34, 53].

4.2.5. Lung function and airway responsiveness (III, IV,V)

Spirometry will be carried out according to the American Thoracic Society criteria[54]. Lung function measurements will be repeated 15 minutes after 400 µg of salbutamol in aerochamber to assess reversibility.

Non-specific bronchial hyper-responsiveness will be measured by methacholine challenge, according to the previous published guidelines. Methacholine will be delivered by inspiration triggered by a dosimeter, five-breath will be needed and PD20 (provocative dose causing a 22% fall in forced expiratory volume in one second (FEV1)) determined.

The capsaicin challenge will be performed accordingly to the previous guidelines [55]. The procedure will be performed in a ventilated area. Fresh dilutions of capsaicin solution (capsaicin in powder (30.54 mg) dissolved in 1 ml of Tween 80, 1 ml of ethanol and 8 ml of normal saline) are performed with physiological saline in the laboratory, previous to the challenge and stored at 4ºC. Dilutions of 0.49, 0.98, 1.95, 3.91, 7.81, 15.63, 31.25, 62.5, 125, 250 and 500 µM are prepared. Three ml of each solution are provided by nebulizer through a dosimeter. Subjects perform a baseline spirometry and then start the protocol: inhaling a control solution of normal saline followed by progressively increasing concentrations of capsaicin solutions at 2 minutes intervals. A breath-activated nebulizer controlled by a dosimeter (Optineb®, Air Liquide), set to nebulize for 0.9s will be used. Subjects are asked to cough freely and the concentrations inducing 2 and 5 coughs during the 30 seconds period after capsaicin administration will be registered. The test is finished when 5 coughs are elicited or when the maximal dose (500 µM) is achieved. At the end of the challenge, a new spirometry is performed.

4.2.6. Allergic and inflammatory systemic markers (III, IV, V)

Skin prick tests will be carried out in accordance international guidelines with a standard battery of commercial extracts for common aeroallergens[56, 57]. Histamine dihydrochloride (10mg/ml) and diluent will be as used as positive and negative controls, respectively. The tested extracts will be stored at +2 to +8ºC when not in use. A small drop of each testing solution will be placed on the volar forearm surface of the patient arm and then pressed against the skin in the centre of the allergen drop for at least 1 s without causing bleeding. Results will be recorded after 15 min. The largest and perpendicular diameter of the wheal for each of the allergens is measured and the following value calculated: largest + perpendicular diameter/2. A subject is defined as atopic in the presence of at least one positive result (regarded if the value calculated was ≥3 mm and controls showed adequate reactions) [56, 57].

Inflammatory systemic markers, namely urine metabolome (study II), will be performed by the same methodology already described in the metabolomic analysis of the exhaled breath condensate on point 4.2.4.

4.2.7. Parasympathetic activity measure (III,IV)

Pupillary measurements will be taken with a portable infrared PLR-200™ Pupillometer (NeurOptics Inc, CA, USA). This comprises in two major parts, a hand-held optical unit and an electronic board. The subjects will be asked to spend at least 15 min in a room to allow their eyes to adjust to the low lighting levels before any measurements are undertaken on their pupils. Each subject will sit upright on a chair and the measurements are performed by the operator by resting the pupillometer optical unit against the subject's face. Two pairs of infrared-emitting diodes of an 880-nm wavelength (programmed to adjust intensity automatically for papillary pigmentation and ambient light) will illuminate the eye. The measurement takes about 3 seconds. At the end of the measurement cycle, a graph of the pupil diameters as a function of time will appear on the host screen. Five pupil light response curves will be recorded. The following parameters will be calculated: initial diameter (Init Dia), minimum diameter (Min Dia), final diameter (Final Dia), time to minimum diameter (TTM), reflex amplitude (RA= Init Dia_Min Dia), constriction velocity (CV), reflex amplitude as percentage of initial diameter (%RRA), maximum constriction velocity (Max CV), time to maximum constriction velocity (TM MCV), maximum redilation velocity (Max RV), time to maximum redilation velocity (TM MRV), relative redilation residue as percentage (%RRR = Init Dia_Final Dia/Init Dia_Min Dia), and time at which pupil has re-dilated 75% of the reflex amplitude (TM 75%).
Heart rate after 15 min rest will be measured , during the procedure verbal instructions and a practice period of paced breathing will be provided.

4.2.8. Meal challenge (IV)

All individuals will be asked to consume two different meals, an high fat/high energy meal and another low fat/low energy. The high-fat/high energy meal will by characterize by: total energy (TE) approximately of 1600kcal, consisted of 40g protein (9,6% TE), 218g carbohydrate (55% TE), and 60g total fat (34% TE) and 18g saturated fat (10% TE). The other meal will be according to the Portuguese guide - Roda dos Alimentos[58], characterized with low-fat/low energy meal: TE approximately with 660kcal (1/3 of 2200kcal), consisted of 50 to 70% TE of carbohydrate, 30% TE of total fat and less than 10% of saturated fat.

4.2.9. Exercise challenge (IV)

Protocol will be performed accordingly as previously published and aimed to access physical fitness, exercise induced bronchoconstriction [59, 60].
Contra-indications to perform exercise testing will be: moderate to severe airflow limitation (predicted FEV1<60% or <1,5L), heart attack or stroke in the last 3 months, uncontrolled hypertension (systolic BP>200 or diastolic BP>100), malignant arrhythmias, know aortic aneurysm, orthopedic limitations to exercise, inability to perform acceptable-quality spirometry, pregnancy, nursing mothers, current use of cholinesterase inhibitor medication (myasthenia gravis).

The challenge will be performed in controlled conditions, ambient temperature between 20-25ºC, with low relative humidity (less or equal to 50%). Heart rate will be monitored during all test by a reliable measurement (three lead electrocardiographic configuration or pulse oximeter), nasal clips will be used during the test and blood pressure will be taken before the challenge.
Exercise challenge will be done in a treadmill with starting with a slope of 5.3% and consists: in a 2 min warm up until the heart rate achieved 70-80% of the estimated HR peak, calculated as (220 – age [years]), (ventilation can also be used to monitor and should reach 40-60% of the predicted maximum voluntary ventilation, estimated by FEV1 x35). The speed will then be adjusted during the first 4 minutes to achieve a workload corresponding to the maximum speed subjects will be able to sustain for the last 4 min, about 95% of the estimated HR. This test usually requires 6 to 8 min, but longer period of time will be needed and will be adjusted to an individual basis and the patient physical condition. At the end of the test VO2, VE, RER, and HR will be measured as well as lung function, namely maximal forced expiratory flow volume loops at 1, 3, 5, 10, 15 and 20 minutes after the challenge. In all patients bronchodilation with salbutamol will be performed after 15 min.

4.2.10. Immune-Endocrine and metabolomic mediators assessment (II, IV, V)

Urine, blood and saliva samples will be collected to assess immune, endocrine and metabolomic assessment markers.

In urine samples metabolomic analysis will be performed with the same protocol described for exhaled breath condensate. But, samples will be collected and immediately frozen at -80ºC, then only those samples that have been related to the outcome, respiratory infections in the swimmers cohort or those that completed all protocol of the clinical trials will be used.

Saliva will be collected as previously published[61], subjects will set quietly and with an initial swallow to empty the mouth, un-stimulated whole saliva will be collected by expectoration into a pre-weighted vial for 2 min with eyes open, head tilted slighty forward and making minimal oro-facial movement and saliva flow rate (ml/min) will be determined by weighing saliva density. Duplicate saliva samples can be analyzed for secretory IgA (S-IgA) using ELISA kit. The S-IgA secretion rate will be calculated by multiplying the S-IgA concentration by saliva flow rate

Blood will be obtained by venepuncture from an antecubital vein and will by collected into two Vacutainer tuber containing EDTA, 7ml and two serum-standard tubes 10 ml. Two of the samples with serum and plasma will be centrifugated and then stored -20ºC. Hematological parameter, total and differential leukocyte counts will be performed in an automated cell-counter. Lymphocyte subpopulations (Cd3, CD4, CD8, CD19, CD56) can be determined by flow cytometry with a three-color flow, in order to enumerate total T cell, T helper, T cytotoxic, B

and NK cells. Side-scatter and forward scatter will be use to gate lymphocyte population by morphology and at least 10 000 lymphocyte events will be acquired per analysis.

Serum Imunoglobulins IgA, IgG, IgM can be measured by nephelometry. Cytokines, IL-1, IL4, IL8 IL-10 also could be measured using commercial ELISA.Insulin, adiponectin, leptin, resistin, tumor necrosis factor-α, serpin/type-1 plasminogen activator inhibitor and interleukin-6 measured by multiplexed bead immunoassay, as previously described[62]. Plasma fatty acids concentration will possibly be analyzed using gas chromatography as previously described[63].

Plasma epinephrine and norepinephrine concentrations might be measured by high-performance liquid chromatography. Plasma adrenocorticotropic hormone concentrations could be measured by an electrochemiluminescence immunoassay while plasma cortisol concentrations radioimmunoassay will be used.

4.2.11. Physical activity measurement and intervention (IV,V)

Physical activity will be estimated by: Baecke questionnaire (**Attachment 8**), and International physical activity questionnaire (**Attachment 9**) and by the mean number of steps/day performed during one week assessed by a pedometer.
Baecke questionnaire(**Attachment 8**) is a reliable and valid instrument and has been previously used in the Portuguese population[64, 65]. It is made up of 16 items that call for a Likert-type response (from 1 to 5), designed to assess different categories of the broad concept of physical activity (work/school, sport and leisure). The work/school index was calculate by eight questions (sat,stood, walke, Lifted heavy loads, sweat, work level. Sport index were reported in 4 questions, as well as leisure time index. Total physical activity score could range from 3 to 15. The International Physical Activity questionnaire (IPAQ) (**Attachment 9**) is a general measure of assessment of physical activity and it complements the Baecke questionnaire in physical activity assessment. It is also a valid and reliable measure is several countries including Portugal and has a comparable validity and reliability to the accelerometer and other questionnaires[66].
IPAQ reports separately vigorous-intensity, moderate-intensity and walking in terms of frequency and duration of each specific type of activity, in the past 7 days. This instrument also reports time spent sitting in an ordinary week day. IPAQ will be an expressed according to the guidelines for data processing and analysis in as MET-min/week (metabolic equivalent). After which they will be classified in three categories, low PA level, moderate PA level and high PA [67] .

Physical activity will be evaluated at baseline by two parameter, a pedometer and accelerometer. The accelerometer (Computer Science Application Actigraph), one of the most widely used accelerometers in physical activity research, uses a uni-axial accelerometer that

measures vertical acceleration and deceleration in 1-min epochs. Participants will put it on the right mid-axillary line of the hip on an elastic belt or clip and wear during one week period and them remove it for sleep or any activity that could cause harm to either the monitor or another person. This can be used to monitor light moderate, hard and very hard activity [68]: light intensity will be considered as <3 metabolic equivalents (METs), moderate 3–5.99 METs, hard 6–8.99 METs, and very hard >8.99 METs. Activity counts cut-off, will correspond to MET levels by and equation (cnts/min=(5.99-1.439008)/0.000795)[68]. Pedometer is also a validated method to evaluate physical activity it will be used in association with an accelerometer for baseline physical activity assessment. To register the number of daily steps the subjects will be instructed to a wear the pedometer placed in the waistline above the knee, as previously published[29] and it will be wear for 1 week, being removed for sleep or other activity that could damage the device, namely bath.

Pedometer will be further used for intervention of reduced physical activity (V). In this study, accordingly to the previous baseline number of steps per day were instructed to reduce daily steps in more than 50%. For that accordingly to the assessed physical activity in baseline evaluation in those that performed >3500steps/day, will be recommended to use elevators instead of stairs, riding in cars instead of walking or bicycling during 2 weeks period. A limit of steps/day will be calculated for each participant and they will be able to see if the limit was achieved. All steps will be recorded in the display and stored in the device for posterior computer download.

4.3. Statistical analysis (I-V)

All statistic analyses will performed using the SPSS 20.0 software (Chicago, Ill) and Excel, Microsoft Office, for data assembly and graphical analysis. Normality of data distribution will be assessed by the Kolmogorov-Smirnov test, histogram and QQ plot distribution. Results will be expressed as median and inter-quartile range or mean and standard deviation and in discrete variables as count and percentage. If a normal distribution is described t-test-student would then be used in continuous variables. For non-normal data, non-parametric tests, namely Mann-Whitney will be used, to compare groups. Measures with discrete distributions are expressed as counts (%) and analyzed with Pearson-Chi Square or Fisher exact test. The minimum threshold for statistical significance will be $p \leq 0.05$. In data with skewed distributions, logarithmic transformation will be done and if appropriate (normal distribution is achieved) comparisons will be made with this change.

4.3.1.Cross-cultural translation and Validation of WURSS (I)

For statistical analysis of questionnaire validation factorial analysis will be used for content validity evaluation using the 7 identified domains of WURSS-21 plus headache, body-ache and fever, which are included in WURSS-24. This approach combines exploratory and confirmatory procedures, using weighted least square estimates employing diagonal weight matrix techniques to seek common factors between the domains; all questioned items will be included in the analysis. Convergent validity will be provided by Pearson correlations between WURSS-21 scores in the first day and SF-36 scores in the third, as this measures health state in the last 4 weeks. For criterion validity sensitivity, specificity and predictive positive values will be calculated, as well as area under the curve. For internal consistency Cronbach`s alpha coefficient in each domain based on the average of scores from the first three days will be calculated and also comparison between the first and last day. To evaluate each day, responsiveness index will be calculated which is equal to minimal important differences (MID)/√(2mean squared error).

<u>Sample size calculation</u>

Sample size was calculated assuming the worst proportion for an item, based in the previous published literature, which was chest congestion, that occurred in a prevalence of 75,7%, then assuming a confidence interval of 0,95% and a α=0,05 (with a standard normal deviate for a two-sided α), the required number would be 280 participants. This sample is adequate for the target factorial analysis in which 24 items will be used (WURSS-21 + headache, body ache and fever). For controls comparison and predictive validity evaluation, assuming that WURSS has a Sensitivity of 0,85 and Specificity of 0,44, and the fact that 70% of the population experiences a cold in a given year[9, 39], for a desired precision of 0,05 for the sensitivity and for a specificity of 0,1 the sample size population needed will be 281 participants. The largest population of 281 patients will be chosen as target for evaluation.

4.3.2. Respiratory Infection susceptibility in athletes and elderly (II,III)

All potential risk factors for respiratory infections will be analyzed for two different in risk populations, elderly and athletes (II,III). Elderly clinical trial will be analyzed as a completed-protocol (III).

Descriptive values for each risk factor will be calculated and Relative Risk to Respiratory Infection with the respective confidence intervals at 95% assessed.

Multiple linear regression and logistic regression models will be used as appropriate to modulate for possible confounders, namely respiratory infections and their severity will be given by questionnaire scores. Univariate and multiple logistic regression models will be developed using independent variables as risk factors for having respiratory tract infections. Results will be presented as odds ratio (OR) with [95% confidence interval (CI)].

Variables potentially included in the model for swimmers illness profile (II) will be: gender, age, BMI (variable categorized), anthropometric changes, history of respiratory tract disease, smoking habits, previous influenza immunization, probiotics consumption, vitamins consumption, sleep quality, anxiety, nutritional status, urinary metabolomic profile, asthma, bronchial hyper-responsiveness, airway inflammation presence and immunological mediators.

Variables used in the elderly intervention model will included: gender, age, BMI, history of respiratory tract disease, diabetes, smoking habits, previous influenza immunization and previous practiced exercise training.

Models will be performed by data driven and progressively adjusted considering its goodness-of-fit (assessed by Hosmer-Lemeshow test) and predictive power (evaluated by ROC curve analysis).

For the moderate exercise intervention trial in elderly (II) sample power was calculated as infrastructure conditions only allowed a maximum sample of 40 persons in each group to be included. Relative risk of having an infection in the training group and in control group will be used. In the previous published literature assessing postmenopausal women [24], having an infection during an moderate-exercise intervention, 48% had at least one episode of respiratory infections compared to control group, which had at 30% in the second. Assuming an alpha of 0.05 on exercise group had 48% change of having infection and 30% of having infections in the non-exercising group the power would be 30%.

<u>Sample size calculation</u>

In the athletes population, all swimmers under competition in the Porto´s team Swimmers will be eligible to the study, a population of 40 participants in total can be evaluated for this study purposes. Using previous studies comparing the rate number of respiratory infections for this specific population, elite swimmers under 12 weeks high intensity period before competitions 40% have a respiratory infection, with an alpha of 0,05 the power will be 35%.

4.3.3. Acute exercise and meal challenge (IV)

In the randomized cross-over study, includes all randomized subjects which took at least one meal challenge and exercise intervention and who had at least one post-baseline immune marker variable measurement. Primary and secondary endpoints will be analyzed for the presence of a carry-over effect in the meal challenge study [69].

Changes in the immune biomarkers and endocrine will be presented as mean ± standard deviation. Data obtained from high fat and low fat meal challenge will be compared using paired t-tests. Repeated-measures analysis of variance (ANOVA) were used to analyze the effects of the challenges.

<u>Sample size calculation</u>

This study is a 2-intervention, 2-period cross over design. Each subject received each meal once in one of the two periods in an order dependent on the randomization, and consequently comparisons were made within-subject. Estimates of between-subject variability have been derived for from previous studies evaluating meal high carbohydrate ingestion in physically active subjects, before an exercise challenge[70]. Standard-deviation was estimated for interleukin-6, more specifically to be 1.3 of standard deviation. This cannot be generalized to this population as they are not physically activ, but no study was previously performed in asthma patients or obese. Only in one previous study performed an high fat challenge in asthma and obese patients and showed a standard deviation in IL-6 of 0.5[63]. Assuming a range difference of 1, it was calculated a value over 80% power to demonstrate (at the 5% level of significance) a difference between interventions of 1 pg/ml in the IL-6, in a sample size of 25 patients in each sequence. To allow for dropouts the study was designed to recruit 30 patients in total, 15 in each group asthma and obese.

4.3.4. Reducing physical activity (V)

The analysis was conducted using the “intention-to-treat” (ITT) approach, including all randomized subjects which took at least one dose of the study preparation/or intervention and who had at least one post-baseline efficacy variable measurement. Changes between baseline and after intervention period was analyzed with paired t-tests. The absence of studies evaluating the immunological outcomes precluded the power analysis.

5. Safety

An Adverse Event (AE) is any untoward medical occurrence in a patient regardless of its causal relationship to the study treatment. An AE can therefore be any unfavorable and unintended sign, symptom or disease, temporally associated with the trial. The occurrence of an AE may come to the attention of study personnel during the study interviews or procedures or upon review by a study monitor. All AEs including local and systemic reactions not meeting the criteria for “serious adverse events” will be registered (event description, time of onset, clinician’s assessment of severity), relationship to study procedures will be searched. All AEs will be followed to adequate resolution. Any medical condition that is present at the time that the subject is screened will be considered as baseline and not reported as an AE. However, if it deteriorates at any time during the study, it will be recorded as an AE. All AEs will be assessed by the clinician using a protocol defined grading system: mild, events require minimal or no treatment and do not interfere with the subject’s daily activities; moderate, events result in a low level of inconvenience or concern with the therapeutic measures and severe, events interrupt a subject’s usual daily activity and may require systemic drug therapy or other treatment. As relationship to the study is concerned an AE will be classified in: associated, event is temporally related to the administration of the study product and no other etiology explains the event and not associated, event is temporally independent of study product and/or the event appears to be explained by another etiology.

A serious adverse event is defined as an AE that meets one of the following conditions: death during the period of protocol-defined surveillance; life-threatening event (defined as a subject at immediate risk of death at the time of the event); inpatient hospitalization or prolongation of existing hospitalization during the period of protocol defined surveillance and any other important medical event that may not result in death, be life threatening, or require hospitalization, but based upon appropriate medical judgment, the event may jeopardize the subject and may require medical or surgical intervention to prevent one of the outcomes listed above.

Safety

No safety issues will come from the participation in the respiratory symptoms survey study (**I**), as only observational data will be retrieved. Data confidentiality will be preserved and the questionnaires will be identified by a number.

For the illness-prone evaluation profile (**II**), baseline assessment procedure as well as measures were previously submitted and accepted by the ethical commission (**Attachment-5**).

For lung function evaluation and testing procedures the following risk and safety measures will be considered:

- Lung function will be assessed by spirometry and bronchodilation response will be evaluated by salbutamol, all good practice guidelines will be followed[54]. These are

safe procedures only a possible discomfort related with forced expiratory maneuvers can be felt, but no adverse reactions are expected.

- In capsaicin challenge in a recent review adverse events were investigated and only minor side effects like throat irritation, self-solved feeling was reported during the procedure [71]. Published contra-indications will be respected[55].
- Methacoline challenge is a standardized procedure widely use for bronchial hyper-responsiveness assessment[60], accordingly to guidelines, a medical trained doctor trained in evaluating and treating bronchospasm will be present at all times and appropriate resuscitation equipment available. Contra-indications published in the guidelines will be respected[60].The procedure will be performed in total safety conditions starting from very small doses and in stepwise, as soon as positive criteria is achieve or any symptom occurs procedure is stopped and will be given inhaled salbutamol to reverse bronchial constriction. Transient symptoms can occur namely dyspnea, cough and chest tightness but will be immediately treated. Participant will be discharged when he returns to their baseline spirometry values and his symptomless.
- Exhaled nitric oxide, exhaled breath condensate measurements were not associated in the literature to safety problems, risks or complications[72].
- Induced sputum is a safe procedure, pre-treatment with salbutamol is used to avoid potential side effects and lung function will be monitored during the procedure with peak expiratory flow, when symptoms occur or a drop occurs in lung function the participant is immediately treated and broncho-constriction reversed. There are no reports of death or need for hospital admission in patients undergoing sputum induction[73].

Puppilometry (II, IV) is a non-invasive method, through an extensive search through databases for “safety”, “risks”, “complications”, and “safety” associated with “pupillometry” retrieved no results concerning potentials risks for this procedure were found. Contraindications, which will be strictly followed, include avoidance when the orbit structure is damaged or surrounding soft tissue has an open lesion or edema.

Blood sampling (II, III, IV and V) will be performed accordingly to the published recommendations and hygiene measures will be followed[74]. Risks related to phlebotomy have been previously described namely infection risk, light-headiness and local hematoma. All potential adverse effects will be registered and oriented by the researcher team. No adverse effects, except the inconvenience will be related with urine and saliva samples collection. The prick-puncture test is safe[57]. Systemic reactions with skin prick tests for inhalant extracts are rare and have decreased dramatically to an overall risk below 0.02% for anaphylactic reactions and no fatalities with aeroallergen commercial extracts have been reported[57]. Mild reactions are expected to occur in 15 minutes (defining a positive test) in a skin prick test local wheal, itching, and erythema. Delayed or systemic reactions are unexpected.

Exercise testing (IV), namely maximal symptom-limited exercise testing is a relatively safe procedure, especially in otherwise healthy individuals. Fatality risk is related to medical complications to the underlying disease and the rate of death per patients during exercise testing is 2 to 5 per 100.000[75]. Strict contra-indications will be followed. A licensed physician and an cardio-respiratory technician will observe the participant during exercise and the recovery period and watch for undue stress (e.g., severe wheezing, chest pain, lack of coordination) or adverse signs (e.g., ECG abnormalities, falling blood pressure, decrease in O2 saturation), if it occurs the test will be immediately stopped and any adverse event treated. A resuscitation cart will be immediately available. The population tested in this study will be young and otherwise healthy without know disease, only chronic co-morbidity will be asthma and obesity. Exercise induced bronchoconstriction is assessed by exercise challenge and it is a recommended and widely used method to evaluate asthma patients. Furthermore these population will be under controlled asthma. If any adverse event occurs, namely bronchoconstriction it will be immediately reversed with bronchodilation.

Exercise intervention in the elderly population (III), was already submitted and accepted by the ethical commission (**Attachment 6**). Moderate exercise program will be performed and adapted to each elderly participant, co-morbidities and difficulties and physical condition and anaerobic resistance for exercise strength will be evaluated previously to the training by resistance maximum. Exercise will then be adapted to each individual condition and progressively increased at each participant rhythm. The exercise will be guided by sports professors trained in exercise teaching and monitoring. Potential adverse conditions can occur, namely muscle and body ache which will be self-limited and prevented by this progressive adaptation to exercise. All elderly will have an individual insurance if any adverse event occurs, namely falls during training or any unavoidable accident.

Physical inactivity intervention (V) was previously studied to evaluate potential endocrinological impact [28-30]. This intervention does not aim to promote inactivity but an reduction of the ambulatory usual activity. During these trials the population was physically active and healthy, as it occurs in the studied population, and no long term adverse events were described. As for immediate side effects, only was seen a reduction of insulin sensitivity, cardiovascular fitness evaluated by VO2 and lean leg mess[28]. No adverse events are expected during the duration of the intervention. At the end of the study a series of recommendations and educational advice will be provided also to the participant which will counteract with any potential adverse events of the well known sedentary behavior. Furthermore, a dietary counseling will be provided by a nutritionist which will also be beneficial for the participant.

High fat meal challenge followed by exercise has not previously been studied in this population. An isolated meal is not associated neither with immediate nor long term adverse events. Meal will be made accordingly to the appropriate hygiene rules and after confection will immediately

be provided to the participant. Only in a previous study high fat challenge increase airway inflammation and reduced bronchodilator recovery, but both of the effects were transitory and no side-effects were reported in the tested asthma and obese population[36].

Unanticipated Problems

Unanticipated problem is any incident, experience, or outcome that meets all of the following criteria: unexpected (in terms of nature, severity, or frequency) given the research procedures that are described in the protocol-related documents; related or possibly related to participation in the research and related with the research places, subjects or others that puts a greater risk of harm (including physical, psychological, economic, or social harm) than was previously known or recognized. Any incident, experience, or outcome that meets the three criteria above, generally will warrant consideration of substantive changes in order to protect the safety, welfare, or rights of subjects or others. Corrective actions or substantive changes that might need to be considered in response to an unanticipated problem include: changes to the research protocol initiated by the investigator prior to obtaining Ethical Commission approval to eliminate apparent immediate hazards to subjects; modification of inclusion or exclusion criteria to mitigate the newly identified risks; implementation of additional procedures for monitoring subjects; suspension of enrollment of new subjects; suspension of research procedures in currently enrolled subjects; modification of informed consent documents to include a description of newly recognized risks; provision of additional information about newly recognized risks to previously enrolled subjects.

All unanticipated problems will be recorded and reported throughout the study.

6. Ethical Considerations

The investigator will ensure that this study is conducted in full conformity with the principles set in the Declaration of Helsinki. The protocol will be submitted for Ethical Commission approval.

Exercise intervention in the elderly population (III), was already submitted and accepted by the ethical commission (**Attachment 6**).
For the illness-prone evaluation profile (**II**), baseline assessment procedure as well as measurements were previously submitted and accepted by the ethical commission in association with the project - Mechanisms of airway damage in elite swimmers (**Attachment 5**).

The investigator will invite the participant to enroll the study participation after explaining the aims and goals of the study. A written information about each study will be provided to the participant (**Attachments V and VI** - presented in the ethical submission dossier). Consent forms and patient information will be given and the subject will be asked to read and review the documents carefully, the participant don`t need to decide at that moment and time will be provide for reflecting about the decision to enroll the study. Upon reviewing the consent form and information, the investigator will explain the research study to the subject and answer any questions that may arise. The subject will then sign the informed consent document prior to any procedures being done specifically for the study. The subjects may withdraw consent at any time throughout the course of the trial. A copy of the informed consent document will be given to the subjects for their records. The rights and welfare of the subjects will be protected by emphasizing to them that the quality of their medical care will not be adversely affected if they decline to participate in this study. No minors will participate in these studies.
Subject confidentiality is strictly held in trust by the participating investigators. This confidentiality is extended to the clinical information relating to participating subjects.
The study protocol, documentation, data, and all other information generated will be held in strict confidence. No information concerning the study or the data will be released to any unauthorized third party. In the event of studies' discontinuation, patients will not be affected.
Confidentiality of the data will be preserved by the investigation groups and the acquired information will only be used for the purposes of this project. Study will end when the time limit for sending the questionnaires is achieved. The final aim of this study will be scientific publication.

Data Handling and Record Keeping

The investigator is responsible to ensure the accuracy, completeness, legibility, and timeliness of the data reported. All source documents should be completed in a neat, legible manner to ensure accurate interpretation of data. When making changes or corrections, cross out the original entry with a single line, and initial and date the change.

Copies of the electronic clinical form will be provided for use as source documents and maintained for recording data for each subject enrolled. Data reported in the electronic clinical form derived from source documents should be consistent with the source documents or the discrepancies should be explained. All source documents and laboratory reports must be reviewed by the clinical team and data entry staff, who will ensure that they are accurate and complete.

Data collection is the responsibility of the clinical trial staff. During the study, the investigator must maintain complete and accurate documentation for the study.

Clinical data (including demographic data, questionnaires, outcome measures, adverse events, concomitant medications, and expected adverse reactions data) as well as laboratory data will be entered into a database in a data system. This data system includes password protection and internal quality checks, such as automatic range checks, to identify data that appear inconsistent, incomplete, or inaccurate.

7. MILESTONES

**I**	**Cross-cultural translation and validation of Wisconsin Upper Respiratory Symptom Survey (WURSS-21) for upper respiratory tract infections monitoring**	Cross-cultural translation and validation of a measuring tool to be applied in upper respiratory tract infection assessment. Validation of a measure that can be further used in respiratory tract infection assessment.
Month 28	Cross-cultural translation concluded	
Month 34	Questionnaire validation	
**II**	**Upper respiratory tract infection susceptibility in athletes – a 3 months follow-up study**	Total number of participants assembled for follow-up procedure. Laboratory analysis will be performed in association with statistic analysis as only the urine that was obtained during an infection period will be analyzed
Month 28	Recruitment and baseline evaluation concluded	
Month 31	Follow-up termination and urine collection	
Month 37	Laboratory analysis and statistical analysis concluded	
**III**	**Effect of physical training on respiratory tract infections in the elderly**	Total number of participants assembled for intervention procedure Laboratory analysis will be done a posteriori and statistical analysis will occur at the same time in order to evaluate which patients had respiratory infections
Month 4	Recruitment and allocation concluded	
Month 10	Exercise program intervention and respiratory symptoms questionnaire assembly	
Month 18	Laboratory and statistical analysis concluded	
**Month 30**	**Study I, II, III publication**	
**IV**	**Effect of an high fat versus healthy diet meal on neuroimmunendocrine response to exercise**	Randomization procedures will be performed alongside the recruitment and baseline achievement. After recruitment intervention can be performed. Both interventions will be done in parallel but independent from one another despite coming from same recruited population
**V**	**Effect of physical activity on immunity**	
Month 39	Recruitment concluded	
Month 39	Randomization to meal and physical activity	
Month 42	Meal challenge followed by exercise intervention concluded	
Month 44	Reducing physical activity intervention concluded	
Month 45	Assessment of safety and adverse events concluded, in order to evaluate long term changes	
Month 50	Laboratory analysis concluded<br>Statistical analysis concluded	
**Month 52**	**Theses presentation, Study IV and V publication**	

8. TIMELINE

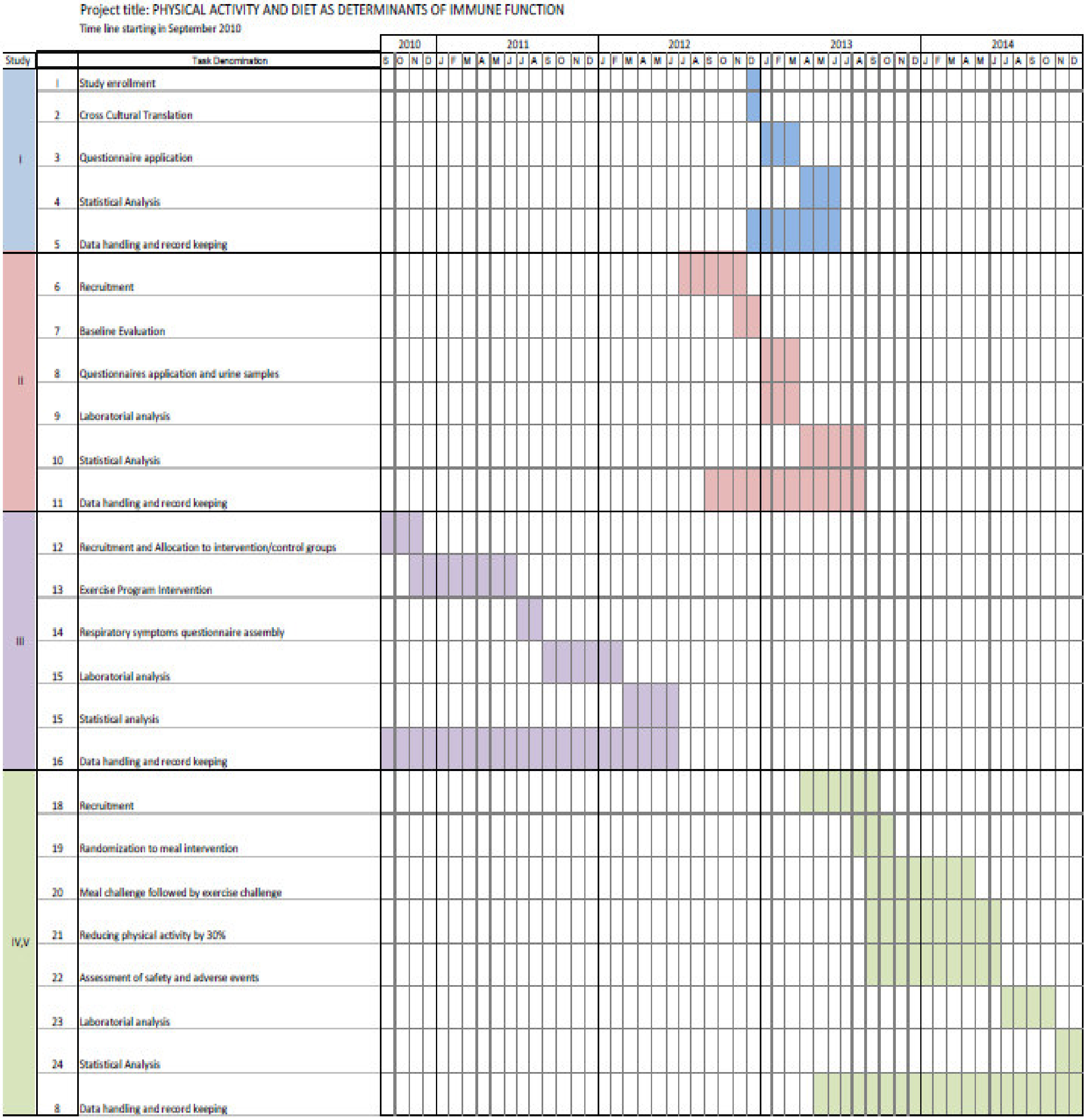

Em anexo ficheiro em excel.

9. HUMAN AND MATERIAL RESOURCES

The research team will be constituted by four physicians, one of those is the main investigator, two nutritionist, who will take part in the nutritional evaluation and food challenge composition, one professor in sports science and one investigator in sports science and physical activity, which will be supporting the physical activity interventions. None of those elements will receive any income from the project participation, but will share publications that come from this project results.

All materials used will be acquired by the study group, namely personal computers and software, letters, stamps and costs of sending the questionnaires to the patients.

For further testing and laboratorial analysis the investigators will apply for funding, until then samples will be stored in proper conditions.

10. EXPECTED RESULTS AND OUTCOMES

Expected results

To obtain a valid method for upper respiratory infections assessment that will permit its monitoring in the clinical and experimental setting.

To evaluate the risk factors related with respiratory infections in a athletes population, namely physical fitness, nutrition and stress showing the multi-factorial side of immunessupression in high intensity exercise.

To assess risk of respiratory infections in a physical activity and inactivity intervention as well as immune mediators impact. Evaluate the interference of high fat diet previously to acute exercise challenge and demonstrate the effect of this association in immune system in a particularly susceptible population (asthma-obese patients). This will permit to understand the effects of exercise in the immune system allowing physicians and trainers to adapt its intensity to specific populations.

Outcomes

Publication of the validation study of a respiratory infections questionnaires that will allow its use in the clinical and experimental setting.

To establish the risk factors for respiratory infection in susceptible populations (elderly, athletes, asthma-obese patients) and to study the immune markers parameter related with acute exercise and physical inactivity interventions (balance between pro-inflammatory and anti-inflammatory cytokines). These will permit the design of a illness-prone profile in the populations studied and a first understanding on the effect of exercise in the immune function.

REFERENCES

1. Walsh, N.P., et al., *Position statement. Part one: Immune function and exercise.* Exerc Immunol Rev, 2011. **17**: p. 6-63.

2. Moreira, A., et al., *Does exercise increase the risk of upper respiratory tract infections?* Br Med Bull, 2009. **90**: p. 111-31.

3. Nieman, D.C., *Exercise immunology: future directions for research related to athletes, nutrition, and the elderly.* Int J Sports Med, 2000. **21 Suppl 1**: p. S61-8.

4. Gunzer, W., M. Konrad, and E. Pail, *Exercise-induced immunodepression in endurance athletes and nutritional intervention with carbohydrate, protein and fat-what is possible, what is not?* Nutrients, 2012. **4**(9): p. 1187-212.

5. Chandratilleke, M.G., et al., *Physical training for asthma.* Cochrane Database Syst Rev, 2012. **5**: p. CD001116.

6. Caspersen, C.J., K.E. Powell, and G.M. Christenson, *Physical activity, exercise, and physical fitness: definitions and distinctions for health-related research.* Public Health Rep, 1985. **100**(2): p. 126-31.

7. Cox, A.J., et al., *Clinical and laboratory evaluation of upper respiratory symptoms in elite athletes.* Clin J Sport Med, 2008. **18**(5): p. 438-45.

8. Jackson, G.G., et al., *Transmission of the common cold to volunteers under controlled conditions. I. The common cold as a clinical entity.* AMA Arch Intern Med, 1958. **101**(2): p. 267-78.

9. Barrett, B., et al., *Validation of a short form Wisconsin Upper Respiratory Symptom Survey (WURSS-21).* Health Qual Life Outcomes, 2009. **7**: p. 76.

10. Murdoch, D.R., et al., *Effect of vitamin D3 supplementation on upper respiratory tract infections in healthy adults: the VIDARIS randomized controlled trial.* JAMA, 2012. **308**(13): p. 1333-9.

11. Gleeson, M., *Immune function in sport and exercise.* J Appl Physiol, 2007. **103**(2): p. 693-9.

12. Kakanis, M.W., et al., *The open window of susceptibility to infection after acute exercise in healthy young male elite athletes.* Exerc Immunol Rev, 2010. **16**: p. 119-37.

13. Pedersen, B.K. and A.D. Toft, *Effects of exercise on lymphocytes and cytokines.* Br J Sports Med, 2000. **34**(4): p. 246-51.

14. Pedersen, B.K. and L. Hoffman-Goetz, *Exercise and the immune system: regulation, integration, and adaptation.* Physiol Rev, 2000. **80**(3): p. 1055-81.

15. Nieman, D.C., *Special feature for the Olympics: effects of exercise on the immune system: exercise effects on systemic immunity.* Immunol Cell Biol, 2000. **78**(5): p. 496-501.

16. Goebel, M.U. and P.J. Mills, *Acute psychological stress and exercise and changes in peripheral leukocyte adhesion molecule expression and density.* Psychosom Med, 2000. **62**(5): p. 664-70.

17. Goebel, M.U., et al., *Interleukin-6 and tumor necrosis factor-alpha production after acute psychological stress, exercise, and infused isoproterenol: differential effects and pathways.* Psychosom Med, 2000. **62**(4): p. 591-8.

18. Nieman, D.C., et al., *Upper respiratory tract infection is reduced in physically fit and active adults.* Br J Sports Med, 2011. **45**(12): p. 987-92.

19. Nelson, M.E., et al., *Physical activity and public health in older adults: recommendation from the American College of Sports Medicine and the American Heart Association.* Circulation, 2007. **116**(9): p. 1094-105.

20. Simpson, R.J., *Aging, persistent viral infections, and immunosenescence: can exercise "make space"?* Exerc Sport Sci Rev, 2011. **39**(1): p. 23-33.

21. Simpson, R.J., et al., *Exercise and the aging immune system.* Ageing Res Rev, 2012. **11**(3): p. 404-20.

22. Kostka, T. and K. Praczko, *Interrelationship between physical activity, symptomatology of upper respiratory tract infections, and depression in elderly people.* Gerontology, 2007. **53**(4): p. 187-93.

23. Woods, J.A., et al., *Effects of 6 months of moderate aerobic exercise training on immune function in the elderly.* Mech Ageing Dev, 1999. **109**(1): p. 1-19.

24. Chubak, J., et al., *Moderate-intensity exercise reduces the incidence of colds among postmenopausal women.* Am J Med, 2006. **119**(11): p. 937-42.

25. Bruunsgaard, H., et al., *Exercise-induced increase in serum interleukin-6 in humans is related to muscle damage.* J Physiol, 1997. **499 (Pt 3)**: p. 833-41.

26. Peake, J.M., et al., *Plasma cytokine changes in relation to exercise intensity and muscle damage.* Eur J Appl Physiol, 2005. **95**(5-6): p. 514-21.

27. Mills, P.J., et al., *Physical fitness attenuates leukocyte-endothelial adhesion in response to acute exercise.* J Appl Physiol, 2006. **101**(3): p. 785-8.

28. Krogh-Madsen, R., et al., *A 2-wk reduction of ambulatory activity attenuates peripheral insulin sensitivity.* J Appl Physiol, 2010. **108**(5): p. 1034-40.

29. Knudsen, S.H., et al., *Changes in insulin sensitivity precede changes in body composition during 14 days of step reduction combined with overfeeding in healthy young men.* J Appl Physiol, 2012. **113**(1): p. 7-15.

30. Olsen, R.H., et al., *Metabolic responses to reduced daily steps in healthy nonexercising men.* JAMA, 2008. **299**(11): p. 1261-3.

31. Mancuso, P., *Obesity and respiratory infections: Does excess adiposity weigh down host defense?* Pulm Pharmacol Ther, 2012.

32. James, K.M., R.S. Peebles, Jr., and T.V. Hartert, *Response to infections in patients with asthma and atopic disease: an epiphenomenon or reflection of host susceptibility?* J Allergy Clin Immunol, 2012. **130**(2): p. 343-51.

33. Adamko, D.J., B.D. Sykes, and B.H. Rowe, *The metabolomics of asthma: novel diagnostic potential.* Chest, 2012. **141**(5): p. 1295-302.

34. Caldeira, M., et al., *Allergic asthma exhaled breath metabolome: a challenge for comprehensive two-dimensional gas chromatography.* J Chromatogr A, 2012. **1254**: p. 87-97.

35. Mamas, M., et al., *The role of metabolites and metabolomics in clinically applicable biomarkers of disease.* Arch Toxicol, 2011. **85**(1): p. 5-17.

36. Wood, L.G., M.L. Garg, and P.G. Gibson, *A high-fat challenge increases airway inflammation and impairs bronchodilator recovery in asthma.* J Allergy Clin Immunol, 2011. **127**(5): p. 1133-40.

37. Barrett, B., et al., *The Wisconsin Upper Respiratory Symptom Survey (WURSS): a new research instrument for assessing the common cold.* J Fam Pract, 2002. **51**(3): p. 265.

38. Barrett, B., et al., *The Wisconsin Upper Respiratory Symptom Survey is responsive, reliable, and valid.* J Clin Epidemiol, 2005. **58**(6): p. 609-17.

39. Barrett, B., et al., *Relations among questionnaire and laboratory measures of rhinovirus infection.* Eur Respir J, 2006. **28**(2): p. 358-63.

40. Barrett, B., et al., *Meditation or exercise for preventing acute respiratory infection: a randomized controlled trial.* Ann Fam Med, 2012. **10**(4): p. 337-46.

41. Ferreira, P.L., *[Development of the Portuguese version of MOS SF-36. Part I. Cultural and linguistic adaptation].* Acta Med Port, 2000. **13**(1-2): p. 55-66.

42. Ferreira, P.L., *[Development of the Portuguese version of MOS SF-36. Part II -- Validation tests].* Acta Med Port, 2000. **13**(3): p. 119-27.

43. Baiardini, I., et al., *Recommendations for assessing patient-reported outcomes and health-related quality of life in clinical trials on allergy: a GA(2)LEN taskforce position paper.* Allergy, 2010. **65**(3): p. 290-5.

44. Yang, S.Y., et al., *Reliability and validity of Wisconsin Upper Respiratory Symptom Survey, Korean version.* J Epidemiol, 2011. **21**(5): p. 313-8.

45. Powell, H., et al., *Validity of the common cold questionnaire (CCQ) in asthma exacerbations.* PLoS One, 2008. **3**(3): p. e1802.

46. Jackson, G.G. and H.F. Dowling, *Transmission of the common cold to volunteers under controlled conditions. IV. Specific immunity to the common cold.* J Clin Invest, 1959. **38**(5): p. 762-9.

47. Zung, W.W., *A rating instrument for anxiety disorders.* Psychosomatics, 1971. **12**(6): p. 371-9.

48. Serra, A., Ponciano, E. & Relvas, J, *Aferição da escala de auto-avaliação de ansiedade, de Zung, numa amostra de população portuguesa – II – Sua avaliação como instrumento de medida.* Psiquiatria Clínica, 1982. **3**(4): p. 203-213.

49. ISAK. *International Standards for Anthropometric Assessment*. 2001; Available from: http://xa.yimg.com/kq/groups/83631355/1318405609/name/6692536-ISAK-BOOK.pdf.

50. Slaughter, M.H., et al., *Skinfold equations for estimation of body fatness in children and youth.* Hum Biol, 1988. **60**(5): p. 709-23.

51. Leone, N., et al., *Abdominal obesity and late-onset asthma: cross-sectional and longitudinal results: the 3C study.* Obesity (Silver Spring), 2012. **20**(3): p. 628-35.

52. Helenius, I.J., et al., *Respiratory symptoms, bronchial responsiveness, and cellular characteristics of induced sputum in elite swimmers.* Allergy, 1998. **53**(4): p. 346-52.

53. Caldeira, M., et al., *Profiling allergic asthma volatile metabolic patterns using a headspace-solid phase microextraction/gas chromatography based methodology.* J Chromatogr A, 2011. **1218**(24): p. 3771-80.

54. Miller, M.R., et al., *Standardisation of spirometry.* Eur Respir J, 2005. **26**(2): p. 319-38.

55. Morice, A.H., et al., *ERS guidelines on the assessment of cough.* Eur Respir J, 2007. **29**(6): p. 1256-76.

56. Heinzerling, L.M., et al., *GA(2)LEN skin test study I: GA(2)LEN harmonization of skin prick testing: novel sensitization patterns for inhalant allergens in Europe.* Allergy, 2009. **64**(10): p. 1498-506.

57. Bousquet, J., et al., *Practical guide to skin prick tests in allergy to aeroallergens.* Allergy, 2012. **67**(1): p. 18-24.

58. Rodrigues, S.S., et al., *A new food guide for the Portuguese population: development and technical considerations.* J Nutr Educ Behav, 2006. **38**(3): p. 189-95.

59. Stensrud, T. and K.H. Carlsen, *Can one single test protocol for provoking exercise-induced bronchoconstriction also be used for assessing aerobic capacity?* Clin Respir J, 2008. **2**(1): p. 47-53.

60. Crapo, R.O., et al., *Guidelines for methacholine and exercise challenge testing-1999. This official statement of the American Thoracic Society was adopted by the ATS Board of Directors, July 1999.* Am J Respir Crit Care Med, 2000. **161**(1): p. 309-29.

61. Gleeson, M., et al., *Respiratory infection risk in athletes: association with antigen-stimulated IL-10 production and salivary IgA secretion.* Scand J Med Sci Sports, 2012. **22**(3): p. 410-7.

62. Martos-Moreno, G.A., et al., *Evaluation of a multiplex assay for adipokine concentrations in obese children.* Clin Chem Lab Med, 2010. **48**(10): p. 1439-46.

63. Wood, L.G., et al., *Improved antioxidant and fatty acid status of patients with cystic fibrosis after antioxidant supplementation is linked to improved lung function.* Am J Clin Nutr, 2003. **77**(1): p. 150-9.

64. Baecke, J.A., J. Burema, and J.E. Frijters, *A short questionnaire for the measurement of habitual physical activity in epidemiological studies.* Am J Clin Nutr, 1982. **36**(5): p. 936-42.

65. Freitas, D., et al., *Tracking of fatness during childhood, adolescence and young adulthood: a 7-year follow-up study in Madeira Island, Portugal.* Ann Hum Biol, 2012. **39**(1): p. 59-67.

66. Craig, C.L., et al., *International physical activity questionnaire: 12-country reliability and validity.* Med Sci Sports Exerc, 2003. **35**(8): p. 1381-95.

67. IPAQ. *Guidelines for Data Processing and Analysis of the International Physical Activity Questionnaire (IPAQ).* International Physical Activity Questionnaire 2005; Available from: http://www.ipaq.ki.se/dloads/IPAQ%20LS%20Scoring%20Protocols_Nov05.pdf.

68. Freedson, P.S., E. Melanson, and J. Sirard, *Calibration of the Computer Science and Applications, Inc. accelerometer.* Med Sci Sports Exerc, 1998. **30**(5): p. 777-81.

69. Hills, M. and P. Armitage, *The two-period cross-over clinical trial.* Br J Clin Pharmacol, 1979. **8**(1): p. 7-20.

70. Robson-Ansley, P., et al., *The effect of carbohydrate ingestion on the interleukin-6 response to a 90-minute run time trial.* Int J Sports Physiol Perform, 2009. **4**(2): p. 186-94.

71. Dicpinigaitis, P.V. and R.V. Alva, *Safety of capsaicin cough challenge testing.* Chest, 2005. **128**(1): p. 196-202.

72. Horvath, I., et al., *Exhaled breath condensate: methodological recommendations and unresolved questions.* Eur Respir J, 2005. **26**(3): p. 523-48.

73. Pizzichini, E., et al., *Safety of sputum induction.* Eur Respir J Suppl, 2002. **37**: p. 9s-18s.

74. WHO, *WHO guidelines on drawing blood: best practices in phlebotomy.*

75. *ATS/ACCP Statement on cardiopulmonary exercise testing.* Am J Respir Crit Care Med, 2003. **167**(2): p. 211-77.

ATTACHMENTS

Attachment 1

Wisconsin Upper Respiratory Symptom Survey-21

Attachment 2

SF-36 v2

Attachment 3

ÍNDICE DE QUALIDADE DE SONO DE PITTSBURGH

Attachment 4

ESCALA DE ANSIEDADE DE AUTO-AVALIAÇÃO DE ZUNG

Attachment 5

Parecer da Comissão de Ética da avaliação basal efetuada para o projeto II (Upper respiratory tract infection susceptibility in athletes – a 3 months follow-up study)

Attachment 6

Parecer da Comissão de Ética da avaliação basal efetuada para o projeto- Effect of physical training on respiratory infections in the elderly/ Infecção Respiratória & Exercício Físico em Idosos)

Attachment 7

Questionários de Controlo da Asma e da Rinite (Asthma Control Questionnaire e CARAT)

**P1** – Durante as **últimas 4 semanas**, quanto tempo é que a asma o/a impediu de fazer as suas tarefas habituais no trabalho, na escola/universidade ou em casa?				
1 – Sempre	2 – A maior parte do tempo	3 – Algum tempo	4 – Pouco tempo	5 –Nunca
**P2** – Durante as **últimas 4 semanas**, quantas vezes teve falta de ar?				
1 – Mais de uma vez por dia	2 – Uma vez por dia	3 – 3 a 6 vezes por semana	4 – Uma a duas vezes por semana	5 – Nunca
**P3** – Durante **as últimas 4 semanas**, quantas vezes os sintomas de asma (pieira, tosse, falta de ar, aperto ou dor no peito) o/a fizeram acordar de noite ou mais cedo do que é costume de manhã?				
1 – 4 ou mais noites por semana	2 – 2 a 3 noites por semana	3 – Uma vez por semana	4 – Uma ou duas vezes	5 – Nunca
**P4** – Durante as **últimas 4 semanas**, quantas vezes usou os seus medicamentos para alívio rápido, em inalador ou nebulizador, como por exemplo salbutamol?				
1 – 3 ou mais vezes por dia	2 – 1 ou 2 vezes por dia	3 – 2 ou 3 vezes por semana	4 – Uma vez por semana ou menos	5 – Nunca
**P5** – Como avaliaria o seu controlo da asma nas **últimas 4 semanas**?				
1 – Não controlada	2 – Mal controlada	3 – Mais ou menos controlada	4 – Bem controlada	5 – Completamente controlada

Attachment 8

Questionário Baecke para avaliação da atividade física habitual

Attachment 9

Questionário internacional de Atividade Física

CES 318-12

No CA c/ parecer favorável DC
25.3.2013

Exmo. Senhor

Presidente do Conselho de Administração do

Centro Hospitalar S. João – EPE

AUTORIZADO
CONSELHO DE ADMINISTRAÇÃO ® REUNIÃO DE 25 MAR 2013
Presidente do Conselho de Administração
(Prof. Doutor António Ferreira)
Directora Clínica (Dra. Margarida Tavares) | Enfermeira Directora (Enfermeira Eurídice Portela) | Vogal Executiva (Dra. [illegible] Barros) | Vogal Executivo (Dr. João Oliveira)

Assunto: Pedido de autorização para realização de estudo/projecto de investigação

Nome do Investigador Principal: Diana Pereira da Silva

Título do projeto de investigação: Programa Doutoral em Investigação Clínica e em Serviços de Saúde - "**PHYSICAL ACTIVITY AND DIET AS DETERMINANTS OF IMMUNE FUNCTION**"

Pretendendo realizar no(s) Serviço(s) de Imunoalergologia do Centro Hospitalar S. João – EPE em colaboração com a Faculdade de Medicina, Nutrição e de Desporto da Universidade do Porto e Unidade Local de Saúde de Matosinhos o projeto de investigação em epígrafe, solicito a V. Exa., na qualidade de Investigador, autorização para a sua efetivação.

Para o efeito, anexa toda a documentação referida no dossier da Comissão de Ética do Centro Hospitalar S. João respeitante a estudos/projectos de investigação, à qual endereçou pedido de apreciação e parecer.

Com os melhores cumprimentos.

Porto, 10 / Dezembro / 2012

O INVESTIGADOR/PROMOTOR

Diana Pereira da Silva

Comissão de Ética para a Saúde do HSJ

Parecer

Projecto de investigação intitulado "Physical activity and diet as determinants of imune function"

Projeto de investigação multicêntrico que se propõe vir a ser desenvolvido no Serviço de Imunoalergologia e Serviço de Imunologia do Centro Hospitalar São João EPE, pela Drª Diana Pereira da Silva, no âmbito do desenvolvimento do programa doutoral do PDICS sob a orientação do Prof. André Moreira.

Do ponto de vista científico, o projeto visa avaliar o impacto da prática de exercício físico e/ou da atividade física e fatores associados como a dieta, na função imune e na susceptibilidade às infeções respiratórias. O estudo prevê ainda: a) validar um questionário de infeções respiratórias para aplicar na observação dos fatores de risco de infeções em populações susceptíveis; b) estudar o efeito de uma refeição calórica vs. normal seguida de prova de exercício no sistema imune, sob a forma de resposta aguda e c) avaliar o efeito da redução da atividade física em marcadores imunológicos, na qualidade de vida e em infeções respiratórias.

São referidos uma série de benefícios do estudo para os participantes, designadamente relacionados com o melhor acompanhamento e a avaliação optimizada de que serão alvo. Como riscos e incómodos invocam-se os decorrentes das deslocações dos participantes e os associados à realização de exames complementares, relativamente aos quais estão clarificadas as condições de segurança em que serão efectuados, designadamente no que respeita à assistência médica.

Serão realizados questionários, dos quais se anexam as respetivas cópias que *não estão anonimizadas em todos os casos.*

A investigadora dispõe da competência científica para a realização do estudo, que está autorizado pelo diretor do Serviço de Imunoalergologia, Dr. José Luis Plácido.

Os dados clínicos dos doentes serão colhidos no respeito pela confidencialidade e anonimato, sem necessidade de FCA.

Está prevista a obtenção de consentimento informado e de uma *informação escrita para o participante, que não foi disponibilizada.*

Prevê-se que os exames complementares a realizar no âmbito do estudo, venham a ser suportados por *financiamento ainda não disponível. O estudo só deverá ser iniciado quando o financiamento for disponível e do mesmo deve ser dado conhecimento à CES.*

Em face da análise do projeto proponho que a sua aprovação pela CES do CHSJ fique dependente da resposta da resposta da investigadora às questões em itálico.

Porto, 20 de Dezembro de 2012

relator

Prof. Manuel Pestana

Face à resposta da investigadora proponho a aprovação do protocolo pela CES do CHSJ 24.01.2013

8. TERMO DE RESPONSABILIDADE

Eu, Diana Pereira da Silva, abaixo-assinado, na qualidade de Investigador Principal, declaro por minha honra que as informações prestadas neste questionário são verdadeiras. Mais declaro que, durante o estudo, serão respeitadas as recomendações constantes da Declaração de Helsínquia (com as emendas de Tóquio 1975, Veneza 1983, Hong-Kong 1989, Somerset West 1996 e Edimburgo 2000) e da Organização Mundial da Saúde, no que se refere à experimentação que envolve seres humanos. Aceito, também, a recomendação da CES de que o recrutamento para este estudo se fará junto de doentes que não tenham participado em outro estudo no decurso do actual internamento ou da mesma consulta.

Porto, 10 / Dezembro / 2012

Diana Pereira da Silva

O Investigador Principal

A Comissão de Ética para a Saúde tendo aprovado o parecer do Relator, aguarda que o Investigador/Promotor esclareça as questões nele enunciadas para que possa emitir parecer definitivo.

2012.12.21 [signature]

Prof. Doutor Filipe Almeida
Presidente da Comissão de Ética

PARECER DA COMISSÃO DE ÉTICA PARA A SAÚDE DO CENTRO HOSPITALAR DE S. JOÃO

emitido na reunião plenária da CES de ___/___/___

Considerados que foram com cuidado os esclarecimentos prestados pelo investigador

A Comissão de Ética para a Saúde APROVA por unanimidade o parecer do Relator, pelo que nada tem a opor à realização deste projecto de investigação.

2013.01.28 [signature]

Prof. Doutor Filipe Almeida
Presidente da Comissão de Ética
